# Evaluation of the published kinase inhibitor set to identify multiple inhibitors of bacterial ATP-dependent mur ligases

**DOI:** 10.1080/14756366.2019.1608981

**Published:** 2019-05-10

**Authors:** Martina Hrast, Kaja Rožman, Iza Ogris, Veronika Škedelj, Delphine Patin, Matej Sova, Hélène Barreteau, Stanislav Gobec, Simona Golič Grdadolnik, Anamarija Zega

**Affiliations:** a Faculty of Pharmacy, University of Ljubljana, Ljubljana, Slovenia;; b Department of Medicinal Chemistry, University of Minnesota, Minneapolis, MN, USA;; c Molecular Structural Dynamics, Theory Department, National Institute of Chemistry, Ljubljana, Slovenia;; d Institute for Integrative Biology of the Cell (I2BC), CEA, CNRS, Univ Paris-Sud, Université Paris-Saclay, Gif-Sur-Yvette Cedex, France

**Keywords:** Bacterial Mur (MurC–MurF) ligases, published kinase inhibitor set, steady-state kinetics measurements, NMR studies, antibacterial agents

## Abstract

The Mur ligases form a series of consecutive enzymes that participate in the intracellular steps of bacterial peptidoglycan biosynthesis. They therefore represent interesting targets for antibacterial drug discovery. MurC, D, E and F are all ATP-dependent ligases. Accordingly, with the aim being to find multiple inhibitors of these enzymes, we screened a collection of ATP-competitive kinase inhibitors, on *Escherichia coli* MurC, D and F, and identified five promising scaffolds that inhibited at least two of these ligases. Compounds **1**, **2**, **4** and **5** are multiple inhibitors of the whole MurC to MurF cascade that act in the micromolar range (IC_50_, 32–368 µM). NMR-assisted binding studies and steady-state kinetics studies performed on aza-stilbene derivative **1** showed, surprisingly, that it acts as a competitive inhibitor of MurD activity towards D-glutamic acid, and additionally, that its binding to the D-glutamic acid binding site is independent of the enzyme closure promoted by ATP.

## Introduction

The increasing emergence of bacterial strains resistant to currently available antibiotics has created medical needs for antibacterial therapy that remain unmet today. It is broadly accepted that new-class agents represent unique and valuable opportunities to achieve significant advances against bacterial resistance, because they should not be as susceptible to the pre-existing mechanisms of resistance, as seen with established antibacterial classes; i.e. they should not show cross-resistance. However, despite large efforts in this area over recent decades, the introduction of mechanistically novel antibacterial agents into clinical practice has been a rarely achieved goal of antibacterial research^1–4^.

**Figure 1.**
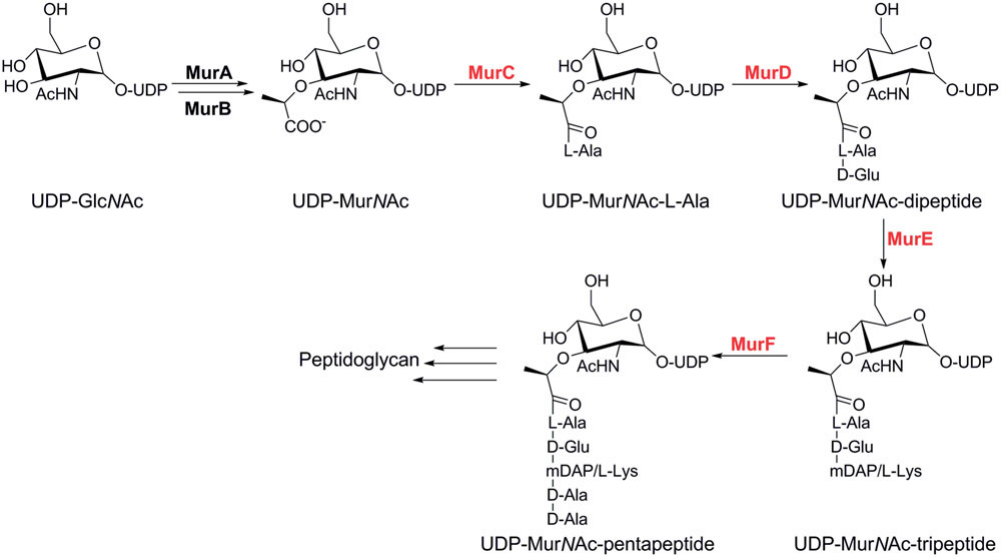
The cytoplasmic steps of peptidoglycan biosynthesis catalysed by the Mur ligases. The Mur ligases considered specifically in the present study are in red.

In this context, there has been increasing interest in exploiting Mur ligases, the enzymes involved in the intracellular steps of the biosynthesis of peptidoglycan, an essential cell-wall polymer unique to prokaryotic cells (Figure 1)^5–7^. Mur ligases catalyse the formation of a peptide or amide bond between a uridine diphosphate *N*-acetylglucosamine (UDP)-substrate and a condensing amino acid. They operate through similar chemical mechanisms, and as shown for MurC and MurF, by an ordered kinetic mechanism^8^^,^^9^. This series of enzymatic reactions is initiated by binding of ATP to the free MurA, followed by binding of the corresponding UDP substrate. The terminal carboxyl group of the UDP substrate is then activated by ATP-promoted phosphorylation, which results in formation of an acylphosphate intermediate. This is then attacked by the amino group of the incoming amino acid or dipeptide, after its binding to MurC. The resulting tetrahedral high-energy intermediate collapses, with elimination of inorganic phosphate and concomitant formation of a peptide or amide bond.

The crystal structures of the Mur ligases from different bacterial strains show a similar three-domain topology, with the N-terminal and central domains binding the UDP precursor and ATP, respectively, while the C-terminal domain binds the condensing amino acid or dipeptide residue. While the topologies of the central and C-terminal domains are similar among the Mur ligases, those of the N-terminal domains show differences, with MurC and MurD more closely related to each other than to MurE and MurF. These differences are associated with the lengths of the UDP precursor substrates. The ATP binding site of the Mur ligases is a highly conserved region, with sequence identities ranging from 22 to 26%. All of the Mur ligases have a glycine-rich P-loop, along with glutamates and histidines, which are responsible for coordinating the Mg^2+^ ions^10^.

The Mur ligase family provides an attractive collection of emerging drug targets, and it has been heavily investigated over the last decade, with novel and diverse classes of inhibitors discovered^11–17^. As all of the Mur ligases considered here (i.e. MurC-MurF) contain the ATP binding site, we screened published kinase inhibitor sets (PKIS) I and II^18^, which are two sets of small-molecule ATP-competitive kinase inhibitors made available by GlaxoSmithKline. Inhibitors that target the ATP binding site of MurC to MurF would have the potential to act as multitarget inhibitors^19^. This constitutes a promising strategy to combat bacterial resistance, because target-mediated resistance to such compounds will be less likely to evolve, as mutations that confer resistance would have to occur to at least two different target genes during a single generation^20^^,^^21^.

However, targeting the ATP binding site of bacterial enzymes is associated with several issues. First, inhibitor binding to the ATP binding site must be selective for the targeted bacterial enzyme over the human ATP-dependent enzymes, and particularly the kinases. Additionally, an ATP-competitive inhibitor must be able to compete with the high ATP concentration in the bacterial cell (0.6–18 mM)^22^, which is remarkably similar to that in human cells (1–10 mM)^23^. Nevertheless, it has been successfully shown that it is possible to design competitive and selective inhibitors of the ATP binding site in protein kinases. Most importantly, successful examples of ATP-competitive bacterial enzyme inhibitors with antibacterial activities and good selectivity profiles with respect to human enzymes show that these challenges can be overcome^20^.

## Materials and methods

### Synthesis of compound 1

**Figure 2.**
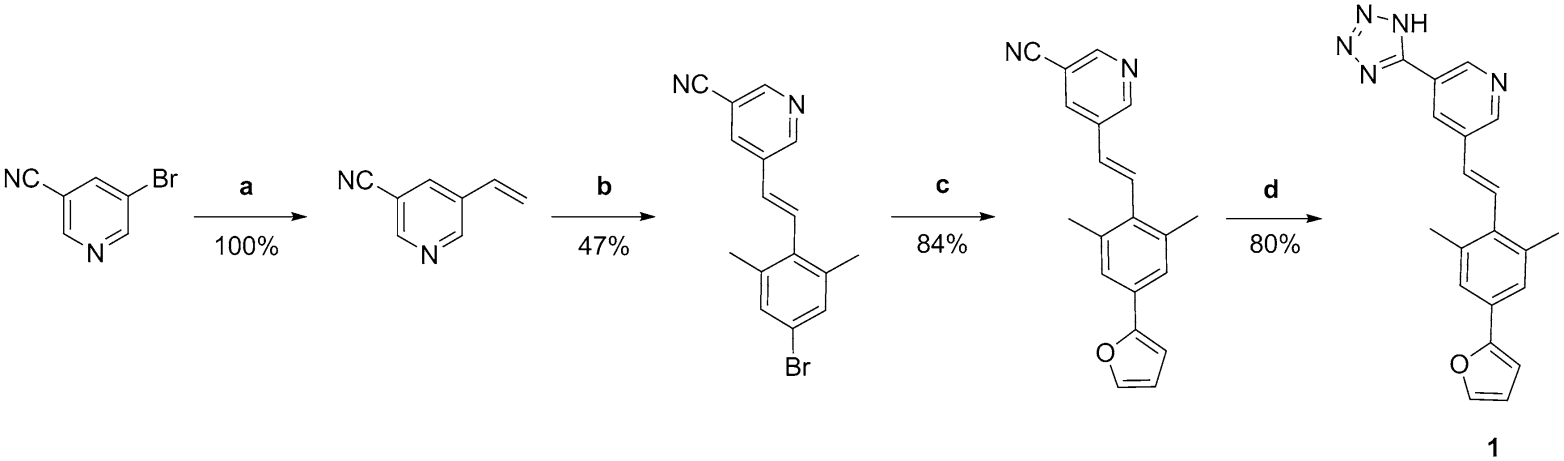
Reaction scheme for synthesis of compound **1**. Reagents and conditions: (a) tributylvinyl tin, LiCl, Pd(PPh_3_)_2_Cl_2_, DMF, 70 °C; (b) 5-bromo-2-iodo-1,3-dimethylbenzene, Pd_2_dba_3_, triethylamine, P(o-tol)_3_, DMF, 95 °C; (c) furan2-ylboronic acid, K_2_CO_3_, Pd(PPh_3_)_4_, water, THF, 100 °C; (d) NH_4_Cl, NaN_3_, anhydrous DMF, 110 °C.

5-bromonicotinonitrile and tributylvinyltin were used in Stille coupling to generate the vinyl intermediate, followed by Heck reaction forming the aza-stilbene derivative. Finally, the tetrazole was synthesised using the NH_4_Cl and NaN_3_ in DMF (Figure 2).

All of the chemicals used were obtained from commercial and were used without further purification. Reactions were monitored using analytical thin-layer chromatography plates (Merck, Silica Gel 60 F_254_, 0.25 mm), and the compounds were visualised with ultraviolet light and ninhydrin. Silica gel grade 60 (particle size 0.040–0.063 mm; Merck, Germany) was used for flash column chromatography. ^1^H and ^13^C NMR spectra were recorded on a Bruker AVANCE III 400 MHz spectrometer in methanol-d_4,_. ESI-MS mass spectra were recorded on the Advion expression compact mass spectrometer (CMS) at the Faculty of Pharmacy of the University of Ljubljana. HPLC analyses were performed on an Agilent Technologies HP 1100 instrument with a G1365B UV–VIS detector (254 nm), using a Luna C18 column (4.6 × 250 mm) at a flow rate of 1 ml/min. In the method the eluent was a mixture of 0.1% TFA in water (A) and acetonitrile (B). The gradient was 10% B to 90% B in 20 min.

*Procedurea*: To a solution of Pd(PPh_3_)_4_ (5 mol%) and LiCl (6.55 mmol) in anhydrous DMF (12 ml), 3-bromo-5-isocyanopyridine (5.46 mmol) and tributyl(vinyl)tin (6.55 mmol) were added under Ar atmosphere and stirred at 70 °C for 16 h. The reaction mixture was cooled to room temperature, followed by addition of saturated solution of KF (20 ml). The formed precipitate was filtered through the pad of Celite and washed with diethyl ether (30 ml). Water phase was extracted with diethyl ether (2 × 40 ml), combined, washed with water (1 × 50 ml), and dried over Na_2_SO_4_. The solvent was evaporated under reduced pressure and the product was purified by flash chromatography.

Procedure *b*: To a solution of 5-vinylnicotinonitrile (5.76 mmol) in anhydrous DMF (12 ml), 5-bromo-2-iodo-1,3-dimethylbenzene (6.92 mmol) was added. Then Pd_2_dba_3_ (10 mol%) and P(*o*-tol)_3_ (10 mol%) were added, followed by triethylamine (1.6 ml). The reaction mixture was stirred at 95 °C for 18 h. To a cooled solution, EtOAc was added (100 ml) and washed with water (2 × 50 ml), 1 M HCl (50 ml), brine (50 ml) and dried over Na_2_SO_4_. The solvent was evaporated under reduced pressure and the formed product was recrystallized from MeOH.

*Procedure c*: To a solution of (E)-5–(4-bromo-2,6-dimethylstyryl)nicotinonitrile (0.96 mmol) in THF (8 ml), water (4 ml) and furan-2-ylboronic acid were added, followed by K_2_CO_3_ (4.78 mmol) and Pd(PPh_3_)_4_ (10 mol%). The reaction mixture was stirred under reflux for 18 h. After cooling to room temperature, EtOAc (20 ml) was added. Organic phase was washed with water (2 × 20 ml) and brine (20 ml) and dried over Na_2_SO_4_. The solvent was evaporated under reduced pressure and the product was purified by flash chromatography.

*Procedure d*: To a solution of (E)-5–(4-(furan-2-yl)-2,6-dimethylstyryl)nicotinonitrile (0.20 mmol) in anhydrous DMF (5 ml), NH_4_Cl (0.60 mmol) and NaN_3_ (0.60 mmol) were added. The reaction mixture was stirred at 110 °C for 48 h. Mixture was cooled to room temperature, then 1M HCl was added dropwise until the white precipitate was formed. Precipitate was filtered off and additionally purified by flash chromatography.

#### Characterisation of compound 1

Yield: 32%, pale yellow crystals, ^1^H NMR (400 MHz, methanol-*d_4_*): δ (ppm) 2.34 (s, 6H, CH_3_), 6.39–6.40 (m, 1H, Ar-H), 6.64 (d, 1H, *J* = 3.4 Hz, Ar-H), 6.70 (d, 1H, *J* = 16.8 Hz, CH), 7.15 (bs, 1H, NH), 7.32 (s, 2H, Ar-H), 7.38 (d, 1H, *J* = 16.6 Hz, CH), 7.42–7.44 (m, 1H, Ar-H), 8.57–8.60 (m, 2H, Ar-H), 8.99–9.00 (m, 1H, Ar-H); 13 C NMR (100 MHz, DMSO-*d_6_*): δ (ppm) 21.6, 106.8, 113.0, 122.4, 123.7, 129.3, 129.7, 131.4, 133.0, 134.9, 135.3, 137.7, 143.6, 145.3, 149.1, 153.5, 154.7; ESI-MS ([*M*–*H*^+^], (*m*/*z*)) calcd. for C_20_H_17_N_5_O: 343.14, found 342.49; HPLC: *t*_R_=12.333 min (99%).

### Enzyme assays for inhibition of Mur ligases

#### Inhibition assay

Inhibition of the Mur ligases was determined using the malachite green assay, with slight modifications^24^. The mixtures for the respective Mur ligase assays had a final volume of 50 µL, which contained 100 µM of each tested compound dissolved in dimethylsulphoxide (DMSO), added to:

MurC: 50 mM Hepes, pH 8.0, 5 mM MgCl_2_, 0.005% Triton X-114, 120 µM L-Ala, 120 µM uridine-5′-diphosphate-*N*-acetylmuramine, 450 µM ATP, and purified *E. coli* MurC^25^.

MurD: 50 mM Hepes, pH 8.0, 5 mM MgCl_2_, 0.005% Triton X-114, 100 µM d-Glu, 80 µM uridine-5′-diphosphate-*N*-acetylmuramoyl-L-alanine (UMA), 400 µM ATP, and purified MurD^26^.

MurE: 50 mM Hepes, pH 8.0, 15 mM MgCl_2_, 0.005% Triton X-114, 60 µM *meso*-diaminopimelic acid, 100 µM uridine-5′-diphosphate-*N*-acetylmuramoyl-l-alanine-d-glutamate, 1000 µM ATP, and purified *E. coli* MurE^27^.

MurF: 50 mM Hepes, pH 8.0, 50 mM MgCl_2_, 0.005% Triton X-114, 600 µM d-Ala- d-Ala, 100 µM uridine-5′-diphosphate-*N*-acetylmuramoyl-L-alanine-D-glutamate-2,6-diaminopimelic acid, 500 µM ATP, and purified *E. coli* MurF^28^.

In all cases, the final concentration of DMSO was 5% (v/v). After incubation for 15 min at 37 °C, the enzyme reaction was terminated by addition of 100 µM Biomol green reagent, and the absorbance was measured at 650 nm after 5 min. All of the experiments were run in duplicate. Residual activities were calculated with respect to control assays without the tested compounds, but with the 5% DMSO carrier. The IC_50_ values were determined by measuring the residual activities at seven different compound concentrations, and they represent the concentration of the compound at which the residual activity was 50%.

#### Steady-state kinetic analysis of compound 1

For compound **1**, K_i_ values were determined against MurD from *E. coli*. K_i_ determinations were performed under similar conditions to those described for the MurD inhibition assay, where different concentration of one substrate and a fixed concentration of the other two were used. First, the concentration of UMA was varied (10, 20, 40, 80 µM) at fixed ATP (400 µM) and D-Glu (100 µM), then the concentration of D-Glu was varied (50, 100, 200, 400 µM) at fixed ATP (400 µM) and UMA (80 µM), and finally, the concentration of ATP was varied (100, 200, 400, 800 µM) at fixed UMA (80 µM) and D-Glu (100 µM). The concentrations of **1** were 0, 15, 30, 60, 90, 120, 180 and 250 µM. After a 15 min incubation, 100 µM Biomol green reagent was added, and the absorbance was read at 650 nm after 5 min. All of the experiments were run in triplicate. The data were analysed using the SigmaPlot 12.0 software. The initial velocities were fitted to competitive, non-competitive, uncompetitive and mixed enzyme inhibition. The *K*_i_ and mode of inhibition from the best ranking model were used, as provided by the software.

### Antimicrobial testing

Antimicrobial testing was performed by the broth microdilution method in 96-well plates following the Clinical and Laboratory Standards Institute guidelines^29^ and European Committee on Antimicrobial Susceptibility Testing recommendations^30^. Bacterial suspensions equivalent to 0.5 McFarland turbidity standard were diluted with cation-adjusted Mueller–Hinton broth with TES buffering (ThermoFisher Scientific), for a final inoculum of 10^5^ CFU/mL. The compounds dissolved in DMSO and the inoculum were mixed together and incubated for 20 h at 37 °C. After this incubation, the minimal inhibitory concentrations (MICs) were determined by visual inspection, as the lowest dilution of the compounds that showed no turbidity. The MICs were determined against *Staphylococcus aureus* (ATCC 29213) and *E. coli* (ATCC 25922) bacterial strains. Tetracycline was used as the positive control on every assay plate, with MICs of 0.5 and 1 µg/mL for *S. aureus* and *E. coli*, respectively.

### Nuclear magnetic resonance studies of ligand binding

The nuclear magnetic resonance (NMR) spectra were recorded at 25 °C on a spectrometer (DirectDrive 800 MHz; Varian, Slovenian NMR centre at National Institute of Chemistry) equipped with a cryoprobe. The pulse sequences provided in the Varian BioPack library of pulse programmes was used. The samples were prepared in 90% H_2_O/10% DMSO-*d*_6_ buffer containing 20 mM HEPES, 7 mM (NH_4_)_2_SO_4_, 3.5 mM MgCl_2_, and 2 mM dithiothreitol, pH 7.2. Here, 0.07 mM MurD that was selectively labelled with ^13^ C at the methyl groups of Ile (δ1 only), Val and Leu was used. The protein was titrated using compound **1** in MurD:ligand molar ratios of 0.5, 1, 2, 4 and 8, without and with 2 mM β,γ-methyleneadenosine 5'-triphosphate (AMP–PCP). At the MurD:ligand molar ratio of 1:8, compound **1** was not completely dissolved. The final concentration of DMSO-*d*_6_ was 12% (*v*/*v*). The variation of DMSO from 10 to 12% (*v*/*v*) affects the chemical shift perturbations by <0.02 ppm^31^.

The heteronuclear single quantum coherence (HSQC) spectra for ^1^H/^13^C were acquired with 1024 data points in *t*_2_, 32 scans, 64 complex points in *t*_1_, and relaxation delay of 1 s. The ^1^H and ^13^C sweep widths were 9470 and 3338 Hz, respectively. The spectra were processed and analysed with the Felix 2007 software package (Felix NMR Inc., Laboratory of Biomolecular Structure at National Institute of Chemistry). The spectra were zero-filled twice and apodised with a squared sine bell function shifted by π/2 in both dimensions using a linear prediction of the data in the incremented dimension. The combined ^1^H/^13^C chemical shift perturbations (Δδ) were calculated from the ^1^H and ^13^C chemical shift perturbations using Equation (1)^32^:

(1) $$\Delta \delta ={\left({\left(\Delta \delta^{1}H\right)}^{2}+{\left(0.252\times \Delta \delta^{13}C\right)}^{2}\right)}^{1/2}.$$

## Results and discussion

### Biological activities

The published kinase inhibitor set compounds were evaluated for inhibition of MurC, D and F ligases from *E. coli* using the Malachite green assay, which detects orthophosphate generated during enzymatic reactions^24^. To avoid non-specific inhibition due to aggregate formation, all of the compounds were tested in the presence of detergent (0.005% Triton-X114). The data are presented as the residual activities of these Mur ligases in the presence of 100 µM of each test compound (Supporting Information Table S1). Compounds that showed residual activity <50% on at least two of these Mur ligases were considered as ‘hits’ (Supporting Information Table S1, gray). The IC_50_ values against MurC, D, E and F were determined for the selected hit compounds – one representative compound from each structural class was chosen: compound **1**^33^, aza-stilbene derivative; compound **2**^34^, alkynyl pyrimidine; compound **3**^35^, pyrazolo [1,5]-b]pyridazine; compound **4**^36^, phenoxypyrimidine; compound **5**^37^, 4,6-*bis*-anilino-1H-pyrrolo[2,3-pyrimidine (Table 1). The four hits (**1**, **2**, **4**, **5**) inhibited all four of these Mur ligases in the micromolar range. Compound **3** only inhibited MurC, and was thus not investigated further. Among the hits, compounds for further mechanistic studies were chosen on the basis of their ligand efficiency. Ligand efficiency quantifies the molecular properties, particularly size and lipophilicity and is used as a selection criterion for structurally different compounds as more ‘efficient’ ligands have the potential to control for the inflation of these properties^38^ Thus ligand efficiencies were calculated for the four hits (Supporting Information Table S2)^39^^,^^40^, and compound **1** was selected as the most promising for additional studies. Additionally, regarding the data of profiled compounds in large panels of human kinase assays^41^ (Supporting Information Table S4), only hit compound **5** among our hits showed appreciable activity at human kinases, thus compound **1** was selected for the further evaluation. To provide sufficient amounts of compound **1** for kinetic and NMR studies, it was resynthesized according to the synthetic procedures (Figure 2) described in the literature^33^.

**Table 1.** Results of *in vitro* biological assays of selected compounds **1**–**5** against *E. coli* MurC–MurF ligases.

			MurC		MurD		MurE		MurF	
Comp.	Name	Structure	RA [%]	IC_50_ [µM]	RA [%]	IC_50_ [µM]	RA [%]	IC_50_ [µM]	RA [%]	IC_50_ [µM]
**1**	GW458344X		70	368	45	104	49	79	26	59
**2**	GW659893X		43	62	51	104	50	157	30	39
**3**	GW827105X		10	74	83	–	84	–	100	–
**4**	SB-242721		48	90	27	63	56	139	51	95
**5**	GSK1173862A		29	62	10	32	26	58	16	66

**Figure 3.**
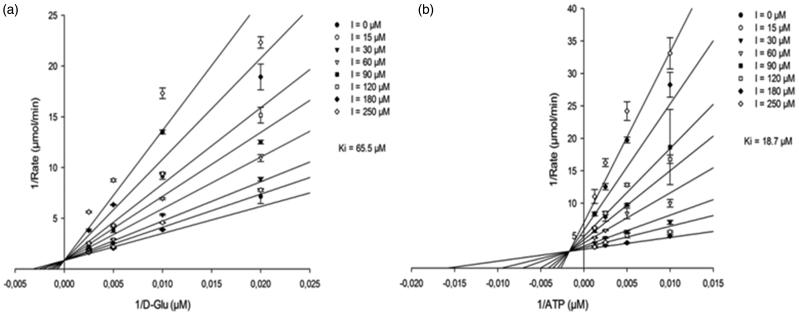
(a) Lineweaver–Burk plot of competitive inhibition model of compound 1 *versus*D-Glu at fixed ATP (400 µM) and uridine-5′-diphosphate-*N*-acetylmuramoyl-L-alanine (80 µM). (b) Lineweaver–Burk plot of mixed inhibition model of compound **1***versus*ATP at fixed d-Glu (100 µM) and uridine-5′-diphosphate-*N*-acetylmuramoyl-L-alanine (80 µM). Data points are means ± standard deviations (all <10%) of triplicates.

To obtain more in-depth insight into the inhibition mechanism of compound **1**, steady-state kinetics and NMR studies of its binding were performed with MurD, the only one of these Mur ligases considered here for which NMR assignation of the crucial methyl groups was available. With implementation of steady-state kinetic measurements, the potential for competitive, non-competitive, uncompetitive and mixed mechanisms of inhibition with respect to each substrate was investigated. The best model obtained showed that compound **1** acts as a competitive inhibitor of MurD with respect to D-Glu (Figure 3(a); Table 2). The K_i_ determined (65.5 ± 4.9 µM) was in good agreement with the IC_50_ for MurD (104 µM) obtained in the standard screening assay. The produced best model of inhibition with respect to ATP showed mixed-type of inhibition (Figure 3(b); Table 2). The model of inhibition versus UMA revealed mixed-type of inhibition, however with less reliable statistics (Table 2; Supporting Information Figure S1).

**Table 2.** Inhibitory properties of compound **1** against all the MurD substrates.

Substrate	Inhibition mechanism	Ki (µM)	*R*^2^
D-Glu	Competitive (full)	65.5 ± 4.9	0.970
ATP	Mixed (full)	18.7 ± 2.6	0.960
UMA	Mixed (partial)	14.8 ± 11.2	0.817

The kinetic data were confirmed with NMR spectroscopic measurements using ^1^H/^13^C-HSQC two-dimensional NMR^42^^,^^43^. The ^1^H/^13^C chemical shift perturbations upon binding of **1** to MurD selectively labelled with ^13^C at the methyl groups of Ile, Val and Leu were examined in relation to the binding of AMP–PCP^44^, UMA^44^ and D-Glu^45^, and of sulphonamide inhibitors^31^^,^^45^ reported previously. Assignments of the crucial methyl groups that indicated binding to the D-Glu binding site (Leu417) in the MurD *C*-terminal domain, or to the uracil binding site (Ile74, Leu57) in the MurD N-terminal domain, were shown in our previous NMR studies of the binding mode of second-generation sulphonamide derivatives^31^. Moreover, we previously identified the signals of the methyl groups that are affected only during the binding of AMP–PCP^44^, which indicated ligand binding to the ATP binding site in the MurD central domain.

The crystallographic structures of MurD complexes^45–47^ offer the understanding of differences in patterns of the ^1^H/^13^C chemical shift perturbations upon binding of various ligands. Namely, the active site for the initial phosphorylation of substrate UMA is located in the cleft between the central and C-terminal domain. The reactive part of substrate UMA enters this cleft from the side closest to N-terminal domain, while the ATP from the opposite site. The sulphonamide inhibitors approach the enzyme from the same side as UMA interacting with all three domains. Their D-Glu moiety occupies the same site in the C-terminal domain as observed for the D-Glu moiety of product UMAG, the naphthalene ring is positioned in the cleft between all three domains, and the functional groups at position 6 of naphthalene ring extends into the binding pocket of the UMA/UMAG uracil ring in the N-terminal domain. Consequently, binding of ATP analogues exert distinct pattern of MurD ^1^H/^13^C chemical shift perturbations in comparison to binding of UMA^44^ or sulphonamide inhibitors^44^^,^^45^. Several pronounced perturbations are observed only upon binding of AMP-PCP. These can be explained by the effect of its adenine moiety, the binding pocket for which is located in the central domain far away from the binding site of UMA and sulphonamide inhibitors. In contrast, several perturbations are observed only at binding of UMA or sulphonamide inhibitors. The most indicative are the large perturbations of Ile74, Leu57 methyl groups in the uracil binding pocket observed upon binding of UMA and sulphonamide derivatives and pronounced perturbation of Leu416 methyl groups in the D-Glu binding site observed upon binding of sulphonamide derivatives. These groups are in the range of 5 Å to the ligand. None of these groups are affected upon binding of AMP-PCP. Moreover, the signals of Leu416 methyl groups are the only ones that are affected upon binding of d-Glu itself^45^. These specific differences in patterns of the ^1^H/^13^C chemical shift perturbations upon binding of various MurD ligands with known binding modes offer a solid basis for identification of binding sites of novel MurD inhibitors.

**Figure 4.**
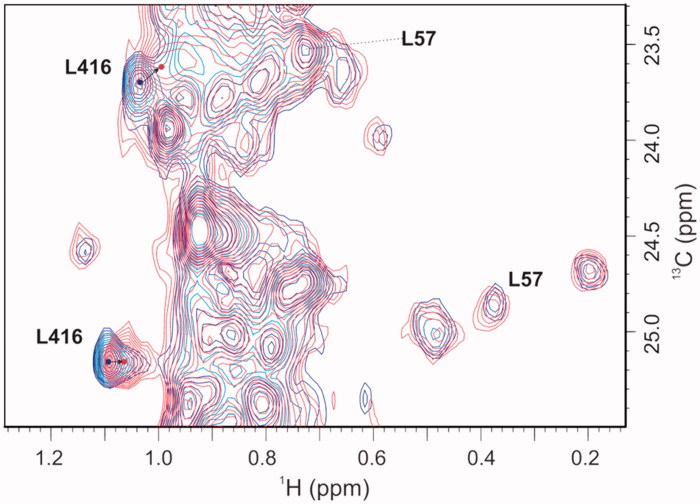
Overlaid expanded regions of the ^1^H/^13^C HSQC spectra for ^13^C selectively-labelled MurD without (blue) and with (ligand:MurD, 4:1; red) compound **1**, which indicates that compound **1** interacts with the D-Glu binding pocket. Both spectra were recorded in the presence of 2 mM AMP-PCP in the NMR sample.

**Figure 5.**
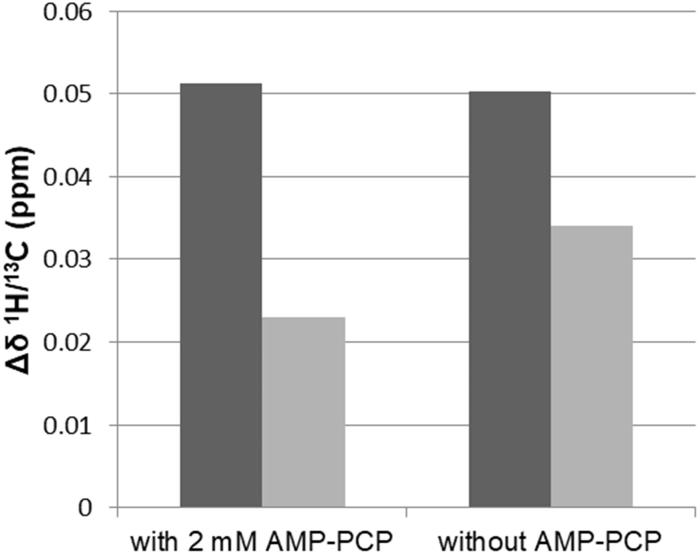
Combined ^1^H/^13^C chemical shift perturbations (Δδ) of higher field (black) and lower field (gray) Leu416 methyl groups, calculated from the ^1^H and ^13^C chemical shifts in the absence and presence of compound **1** at a ligand:MurD ratio of 4:1. Δδ of Leu416 methyl groups upon addition of compound **1** in the presence of 2 mM AMP–PCP (right) and without any addition of AMP–PCP (left) are presented.

Compound **1** only perturbed the signals of the Leu416 methyl groups, as shown in the expansion of the ^1^H/^13^C HSQC spectra with the signals of the crucial Leu methyl groups in Figure 4. The signals of the Leu57 methyl groups that are indicative for the location of a ligand in the uracil binding pocket were unperturbed (Figure 4). These observations indicated that the location of compound **1** was in the D-Glu binding pocket. The combined ^1^H/^13^C chemical shift perturbations of the Leu416 methyl groups upon addition of **1** were similar in the absence and presence of AMP–PCP (Figure 5). Moreover, regardless of the AMP–PCP concentration in the NMR samples, none of the signals of methyl groups that were affected only during the binding of AMP–PCP were perturbed upon addition of **1**. This indicates that **1** does not interact with the ATP binding site in the MurD central domain. It approaches the enzyme from the other side as ATP, in a manner similar to D-Glu or sulphonamide inhibitors. In contrast to sulphonamide inhibitors, it lacks strong interactions with the uracil binding pocket in the N-terminal domain.

The PKIS library consists of kinase inhibitors that target ATP binding sites. Thus, considering that compounds **1**, **2**, **4** and **5** show multiple inhibition of all four of these Mur ligases (i.e. MurC–MurF), we anticipated these compounds bind to the ATP binding site of these Mur ligases. However, the steady-state kinetic measurements together with the NMR studies of ligand binding indicated that aza-stilbene derivative **1** binds to the D-Glu binding site of MurD ligase. Additionally, the observation that binding of **1** is independent of AMP-PCP indicates that the mechanism of MurD regulation^48^ still needs to be defined.

The scaffold of **1** should represent a promising starting point for development of potent MurD inhibitors. This also arises from the possible negative influence of the MurD fast domain motion on ligand binding. It was shown that binding of sulphonamide inhibitors^31^ that span from the C-terminal to the N-terminal domains is exposed to the rapid bending-like motion of the C-terminal and *N*-terminal domains. Therefore, development of inhibitors that interact with either the *C*-terminal or *N*-terminal domain might represent a future perspective.

Selected hit compounds **1–5** were assayed *in vitro* for their potential antibacterial activities against the Gram-negative *E. coli* and Gram-positive *S. aureus* bacterial strains. These compounds were inactive against all of the bacteria strains tested. This was, however, as expected, due to their relatively low inhibitory activities.

Additionally, on the basis of the structures of 11 analogues (Supporting Information Table S3) of compound **1** from the PKIS set, simple SAR can be established which could offer the basis for the further development of more potent (and selective) aza stilbene inhibitors of Mur ligases.

## Conclusions

In summary, screening of the PKIS yielded four new scaffolds that show potential for development into potent inhibitors of bacterial cell-wall biosynthesis. For one of the hits, the aza-stilbene derivative **1**, the binding mode for MurD was defined, and these data represent a good starting point for structural optimisation of Mur ligase inhibitors which contain this scaffold. We showed that compound **1** binds to the D-Glu binding site in the C-terminal domain of MurD independent of AMP-PCP. This is an interesting example that indicates that the conformational states of MurD need to be additionally evaluated along with ligand binding. Also, as inhibitor **1** interacts with the single C-terminal domain, its binding is not affected by rapid movement of MurD domains, which might aid the process towards development of more potent compounds. For an insight into the binding of other identified structural types that would provide data for development of more potent Mur ligase inhibitors, further kinetic and NMR/crystallographic studies should be performed.

Here, it is worth noting that we are aware that the use of protein kinase inhibitors that target the ATP binding site as hit compounds for bacterial enzymes is challenging because of their lack of specificity. However compounds identified as hits (**1**,**2**,**4**) showed no appreciable activity at human kinases^41^ (Supporting Information Table S4) and therefore are a viable starting point for development of bacterial cell wall synthesis inhibitors. Moreover, a bioactivity search for all hit compounds (**1**–**5**) in ChEMBL database^49^ was performed (Supporting Information Table S4). Aside from results of PKIS set profiling on panels of human kinase assays, these data show no activity on other human targets. An approach that can also be used for the design of selective inhibitors of the Mur ligases is the structure-based drug design of compounds that not only occupy the binding site, but at the same time exploit interactions with amino acids adjacent to the binding domain that are unique to these target enzymes. Further studies will also have to concentrate on the design of molecules that will on the one hand fulfil the pharmacophore pattern, and on the other hand have appropriate physical–chemical properties that will allow efficient compound penetration into bacterial cells, which will promote increased antibacterial activity.

## Supplementary Material

Supplemental Material

Supplemental Material
