# Supplementary material for: Evaluation of the published kinase inhibitor set to identify multiple inhibitors of bacterial ATP-dependent mur ligases
Source: J Enzyme Inhib Med Chem. 2019 May 10;34(1):1010–7. doi: 10.1080/14756366.2019.1608981 (PMC6522912; doi:10.1080/14756366.2019.1608981)
Supplement: Supplemental Material [file IENZ_A_1608981_SM5673.pdf]

Evaluation of the published kinase inhibitor set to identify multiple inhibitors of bacterial ATP-dependent Mur ligases

Martina Hrast[a], Kaja Rožman[a,b], Iza Ogris[c], Veronika Škedelj[a], Delphine Patin[d], Matej Sova[a], Hélène Barreteau[d], Stanislav Gobec[a], Simona Golič Grdadolnik[c], Anamarija Zega[a]*

[a]Faculty of Pharmacy, University of Ljubljana, Aškerčeva 7, 1000 Ljubljana, Slovenia

[b]Department of Medicinal Chemistry, University of Minnesota, 308 Harvard Street Southeast, Minneapolis, Minnesota 55455, USA

[c]Laboratory of Biomolecular Structure, National Institute of Chemistry, Hajdrihova 19, 1001 Ljubljana, Slovenia

[d]Institute for Integrative Biology of the Cell (I2BC), CEA, CNRS, Univ Paris-Sud, Université Paris-Saclay, 91198 Gif-sur-Yvette cedex, France

***Corresponding author: Anamarija Zega**

Faculty of Pharmacy

University of Ljubljana

Ljubljana, Slovenia

Tel: +386-1-4769673

Fax: +386-1-4258031

Supporting information

Table of contents:

1. Results of in vitro biological assays of PKIS set compounds against E. coli MurC, MurD and MurF ligases
2. Ligand efficiency values for hit compounds
3. Enzyme kinetic graph
4. Data of profiling in large panels of human kinase assays
5. Inhibitory activities of analogs of the compound 1
6. ChEMBL bioactivity search for hit compounds
7. Reference

1. Results of in vitro biological assays of PKIS set compounds against *E. coli* MurC, MurD and MurF ligases

All the compounds were soluble in 100% DMSO, and at 100 µM concentration in the assay mixtures. In the initial screening compounds were assayed on 96-well microtiter plates at one concentration against three Mur ligases, MurC, D and F.

Table S1: Results of in vitro biological assays of PKIS set compounds against *E. coli* MurC, MurD and MurF ligases

Substance ID	Structure	RA [%] at 100 µM		
		MurC	MurD	MurF
GW786460X	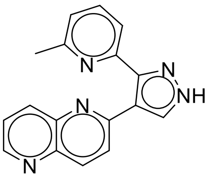	82	76	91
GW680975X	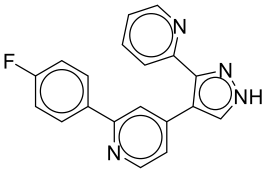	68	84	96
GW679410X#4	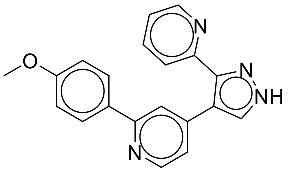	61	98	82
GW695874X	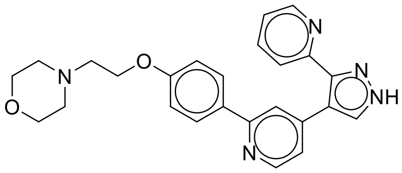	79	80	91

GW682841X	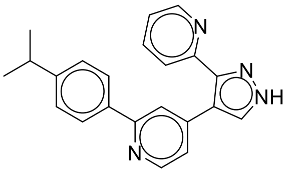	78	85	88
GW711782X	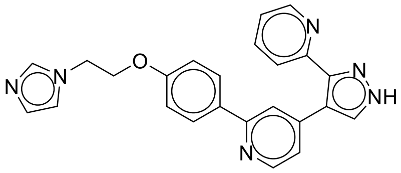	100	74	93
SKF-86055	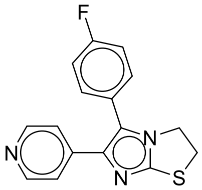	98	91	89
SKF-86002-A2	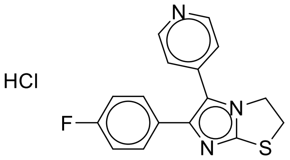	75	91	97
GI98581X#3	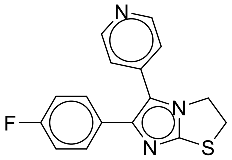	81	nd	54
GW301789X	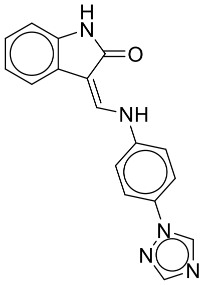	64	91	75
GW416981X	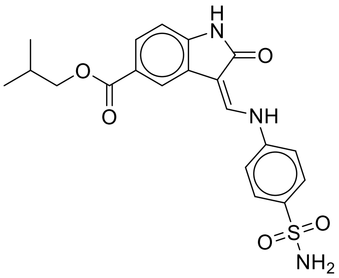	60	100	67
GW278681X	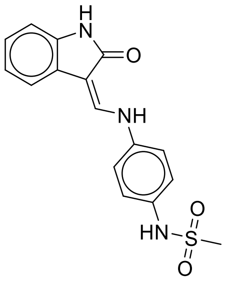	71	89	77

GW275616X	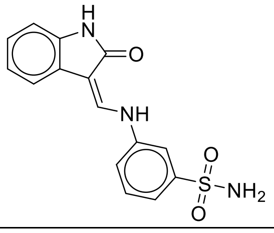	84	88	83
GW416469X	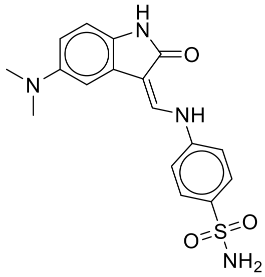	76	89	79
GW297361X	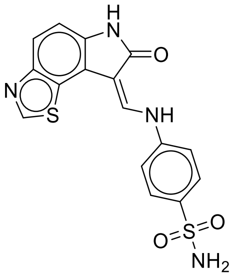	107	76	86
GW335962X	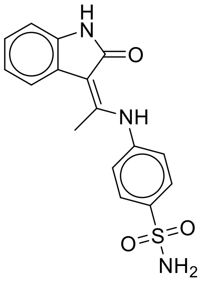	72	76	88
GW284408X	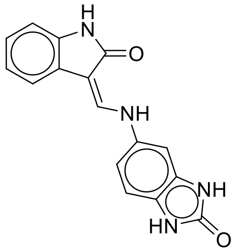	105	86	86
GW407323A	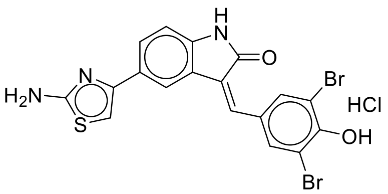	64	54	41
GW441756X	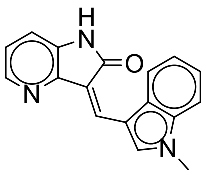	75	99	89

GW405841X	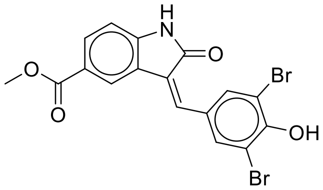	93	82	75
GW429374A	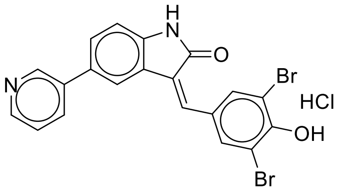	30	51	16
GW406108X	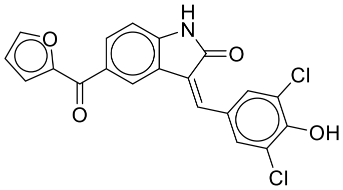	74	85	79
GW442130X	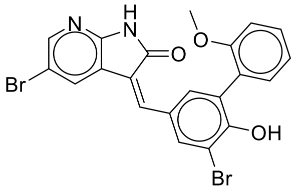	100	84	67
GW305074X	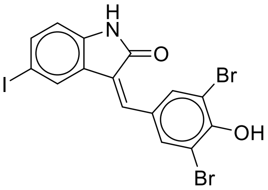	100	92	78
GR105659X	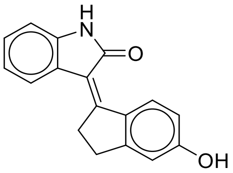	85	100	100
GW279320X	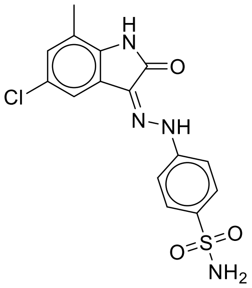	76	78	0
GW305178X	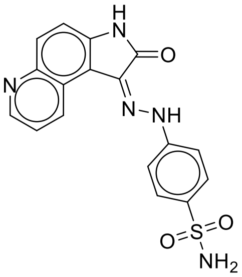	52	57	56

GW290597X	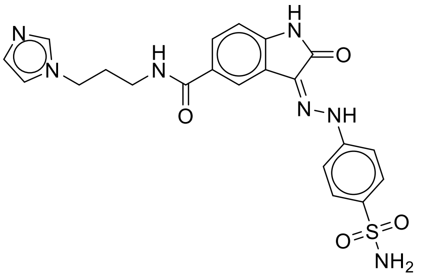	63	92	100
GW300660X	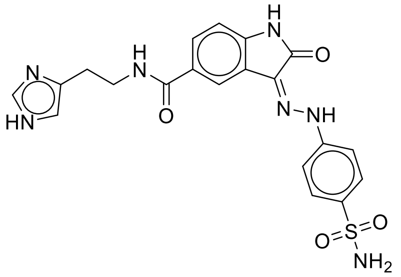	58	90	96
GW300657X	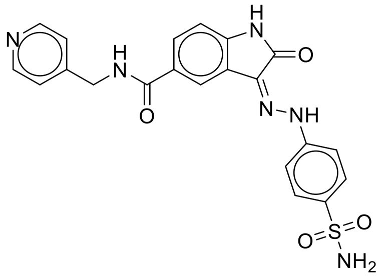	75	96	85
GW300653X	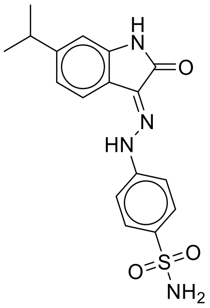	100	85	75
GW282536X	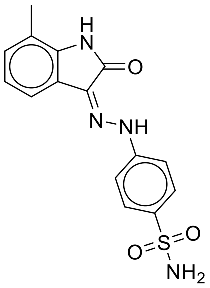	93	nd	27
GW352430A	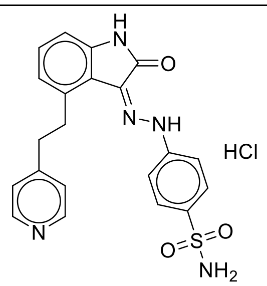	64	90	37

GW275944X	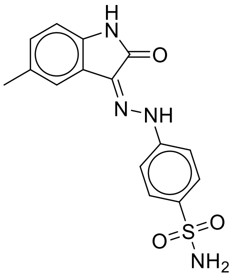	92	87	83
GW276655X	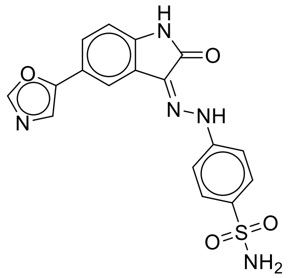	84	73	22
GW280670X	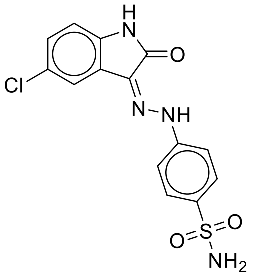	100	99	76
GW576609A	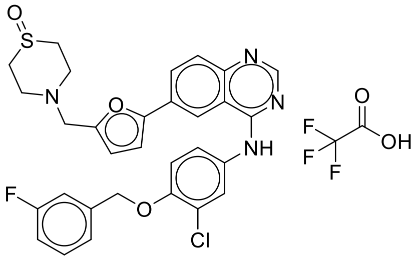	71	54	78
GW680191X	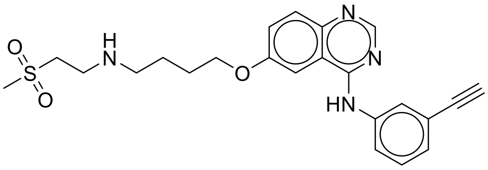	75	93	102
GW580496A	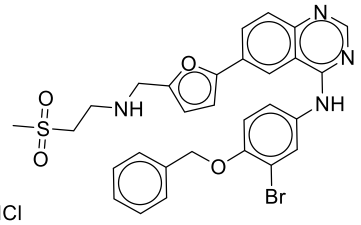 HCl	95	48	90
GI261520X#4	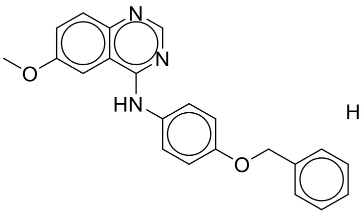 HCl	50	nd	63

GW282449A	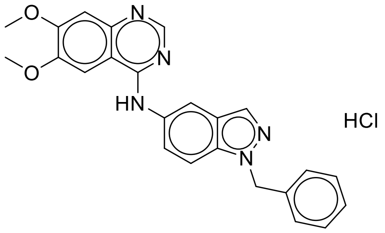	53	62	56
GW574783B	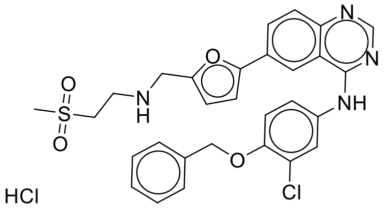	58	37	79
GW576609B	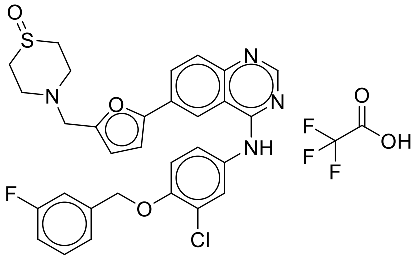	57	nd	84
GW284372X	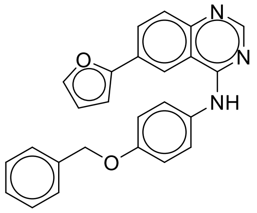	56	80	100
GW621823A	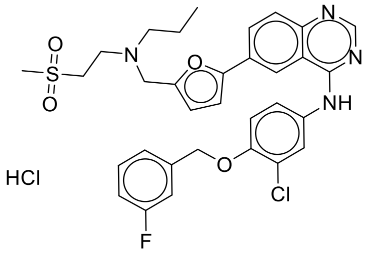	36	42	96
GW567808A	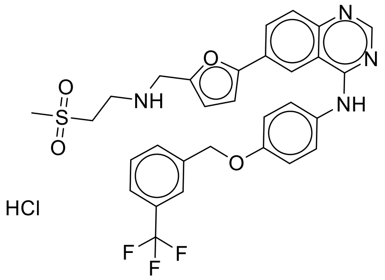	56	34	100
GW410563A	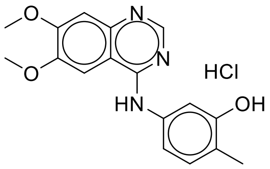	60	92	100

GW576924A	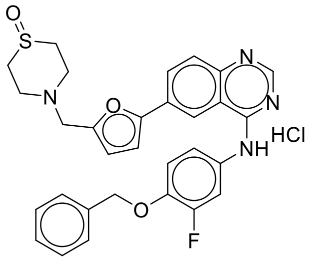	39	85	58
GW576484X	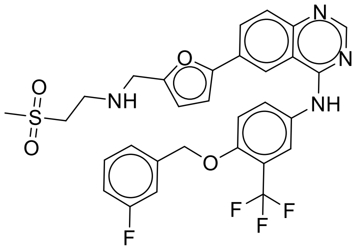	100	55	100
GW574782A	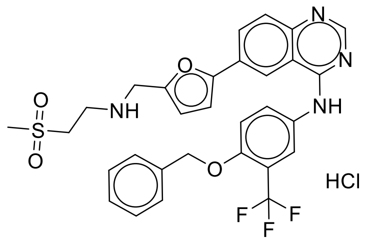	83	60	100
GW568377B	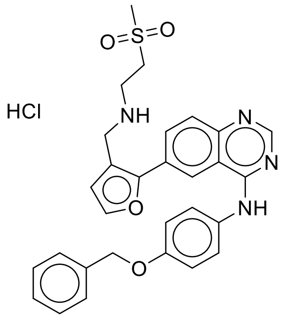	65	68	56
GW633459A	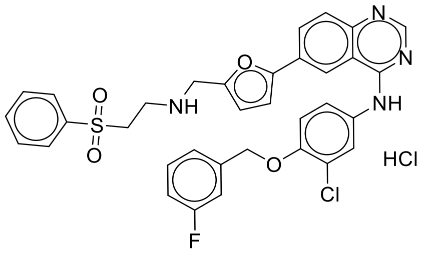	100	80	84
GW458787A	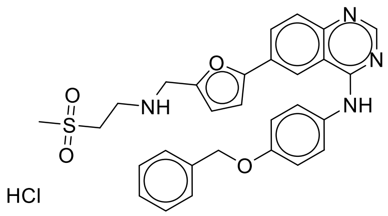	68	58	66
GW616030X	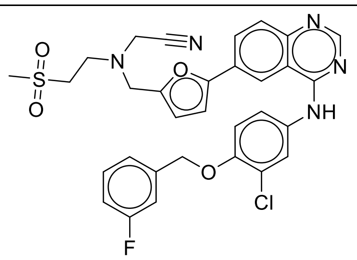	76	59	80

GW566221B	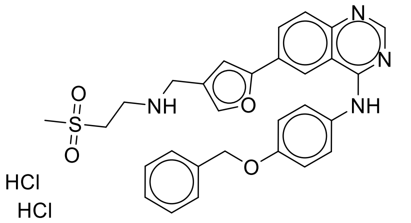	69	69	82
GW461104A	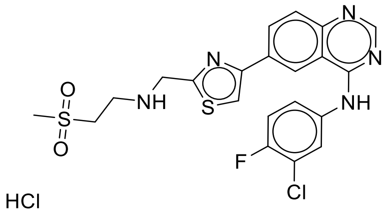	88	73	84
GW583373A	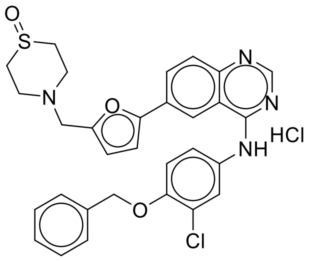	62	59	60
GW615311X	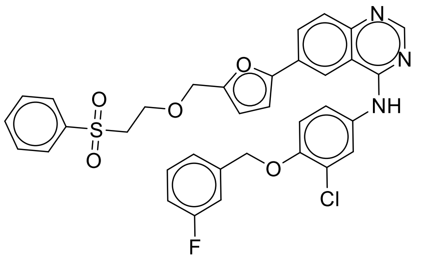	97	91	100
GW458787B	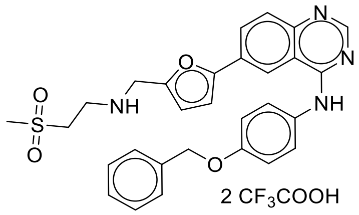	81	nd	71
GI261520A	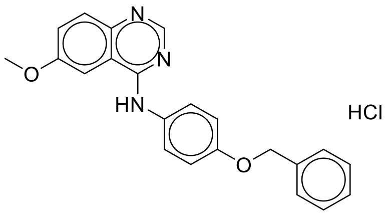	58	31	81
GR269666A	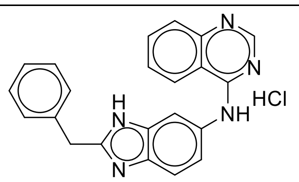	77	83	96
GW301888X	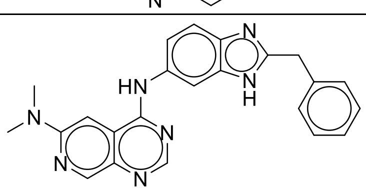	83	80	75

GW282974X	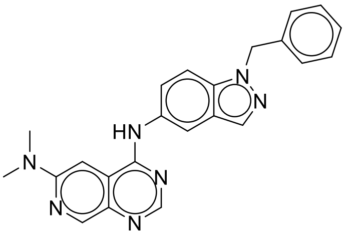	77	70	55
GW440139B	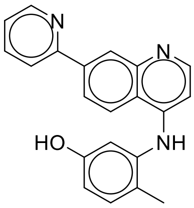	73	73	66
GSK3206866A	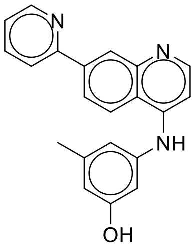	76	nd	29
GW559768X	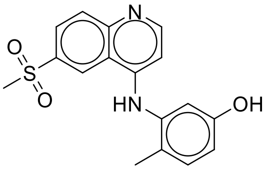	80	93	79
GSK980961A	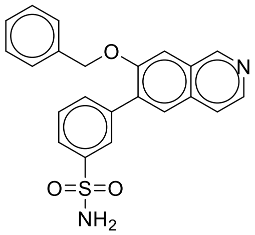	80	86	66
SB-759335-B	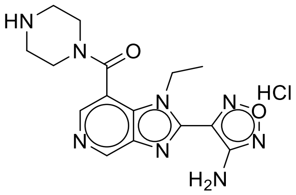	83	nd	94
SB-693162	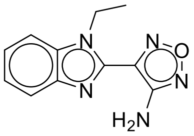	78	nd	100
SB-737198	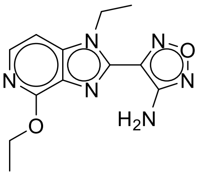	73	100	100
SB-751148	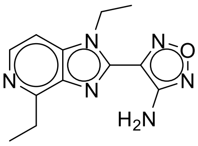	75	96	88

SB-736290	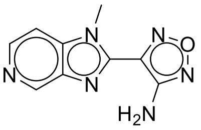	71	nd	90
GSK554170A	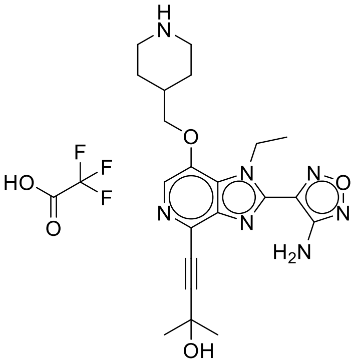	85	89	96
SB-751399	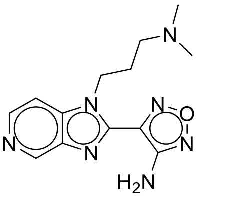	84	93	94
GSK619487A	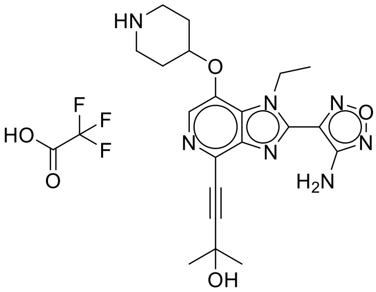	77	86	100
SB-734117	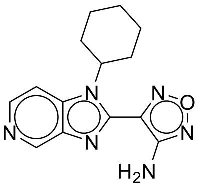	100	79	93
GW549034X	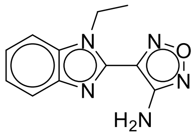	70	100	93
SB-738561	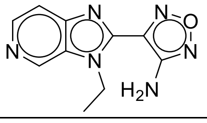	78	58	100
GSK614526A	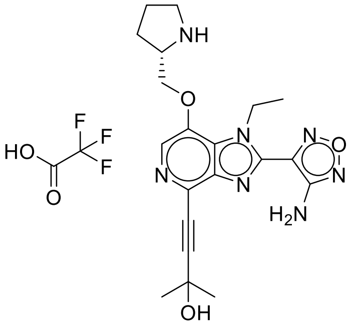	65	89	100

GSK938890A	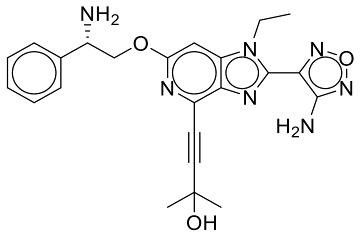	76	80	92
GSK561866B	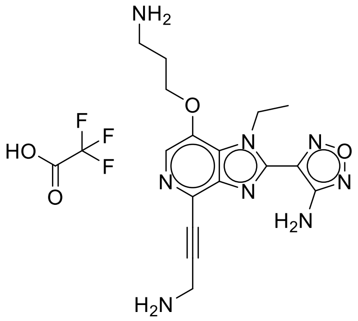	52	82	66
GSK949675A	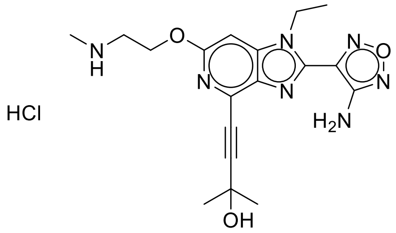	73	85	74
SB-736302	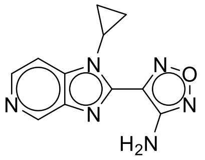	92	100	77
SB-772077-B	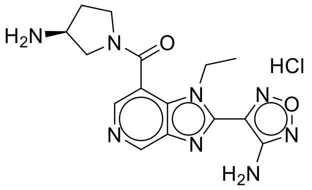	77	90	80
GW785404X	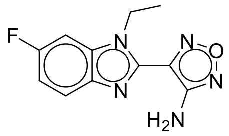	100	88	79
GSK269962B	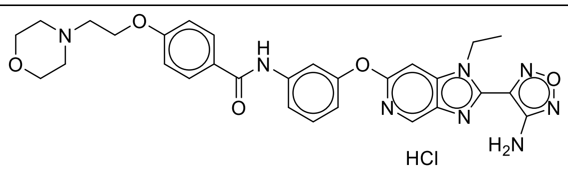	57	58	66
SB-285234-W	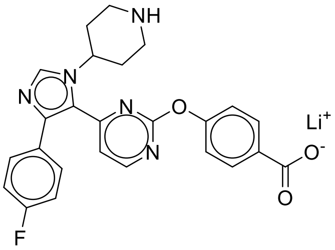	72	61	87

SB-278538	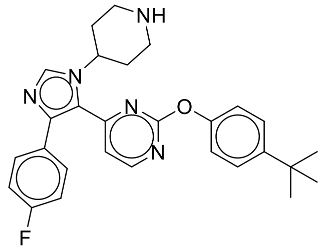	75	57	73
SB-242718	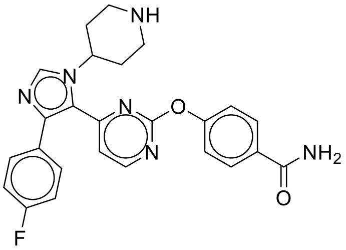	39	65	69
SB-253228	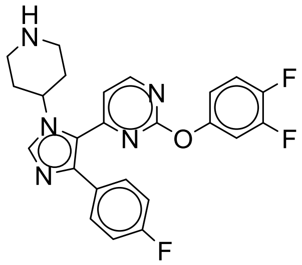	32	40	70
SB-220025-R#3	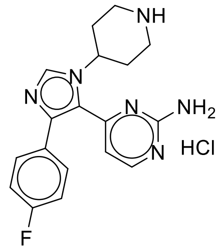	81	87	100
SB-223133	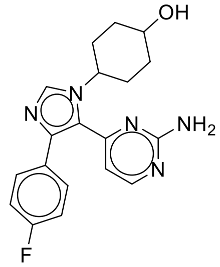	64	87	88
SB-245392	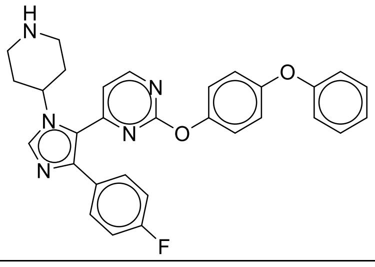	27	41	41
SB-251527	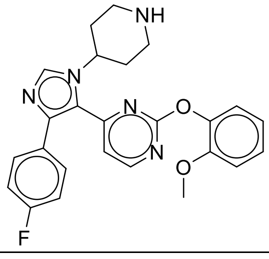	58	71	74

SB-242721	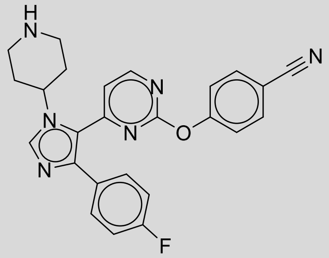	48% IC_{50} = 90 µM	27 IC_{50} = 63µM	19 IC_{50} = 95 µM
SB-254169	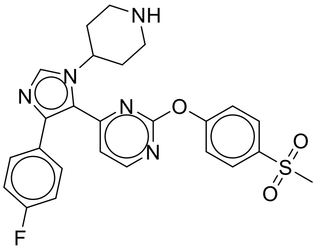	18	49	48
SB-220455	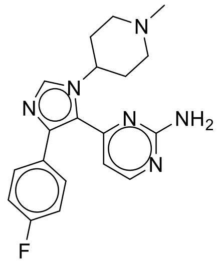	76	81	100
SB-226879	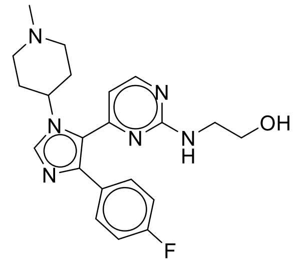	65	98	68
SB-242719	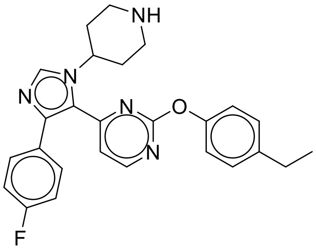	51	87	80
SB-250715	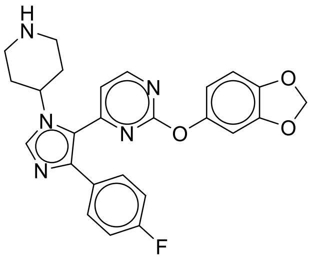	51	65	85
SB-278539	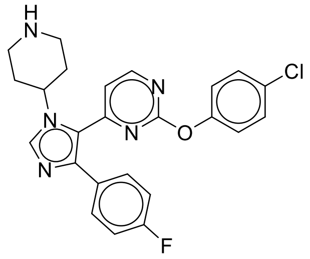	55	52	74

SB-221466	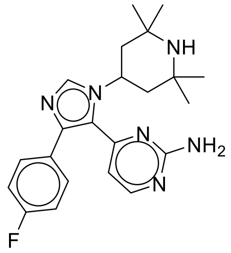	91	93	100
SB-264865	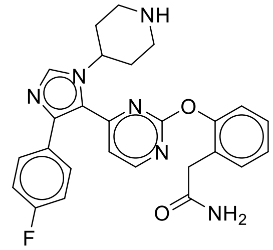	66	82	98
SB-242717	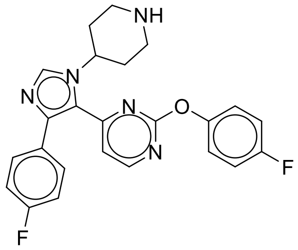	79	100	72
SB-236687	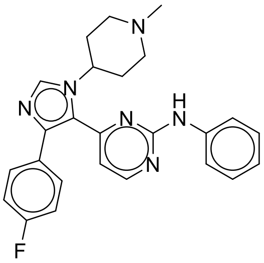	79	90	67
SB-220025-A	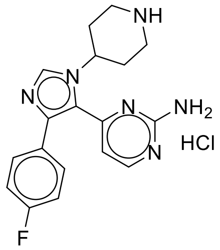	98	99	96
SB-239272	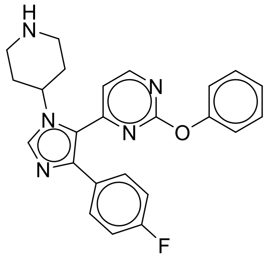	100	67	75
SB-264866	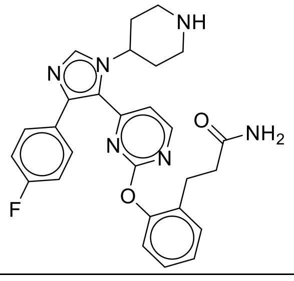	74	83	84

SB-253226	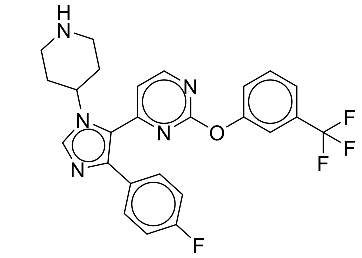	42	24<br>IC_{50} =<br>208<br>μM	69
SB-216385	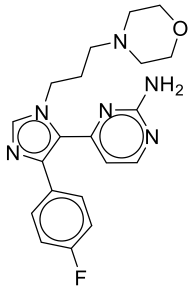	77	96	98
SB-210313	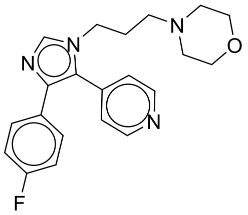	100	91	100
SB-633825	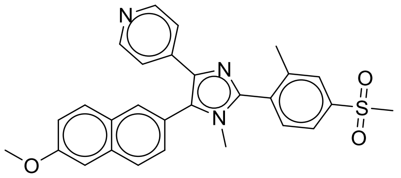	36	69	56
SB-437013	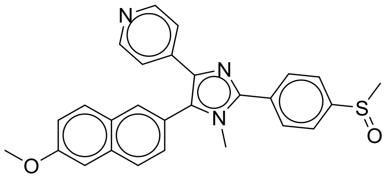	70	58	70
SB-431542-A	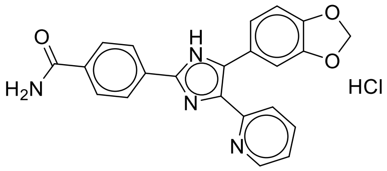	59	90	100
SB-614067-R	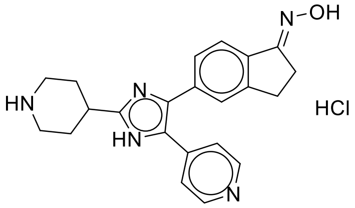	74	100	100
SB-476429-A	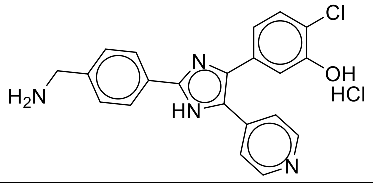	90	55	84

SB-610251-B	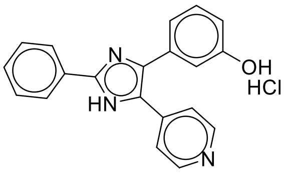	74	13	96
SB-590885-AAD	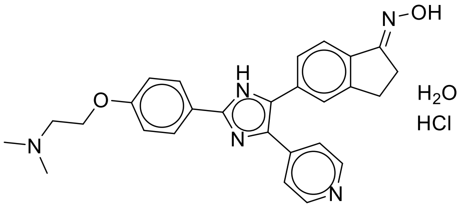	74	100	100
SB-431533	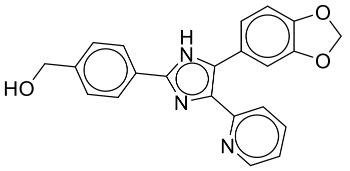	95	nd	75
SB-682330-A	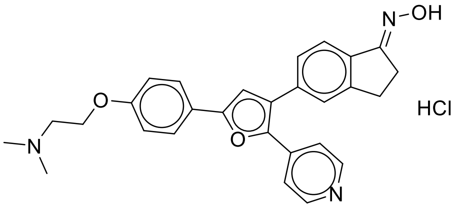	31	51	57
GW780159X	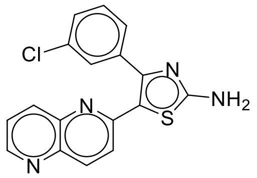	56	90	83
GW693481X	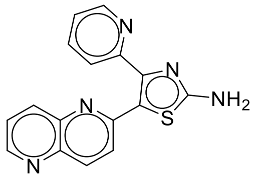	99	88	94
GW785804X	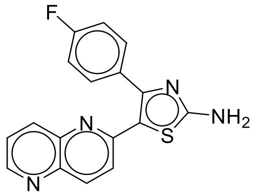	100	82	78
GSK200398A	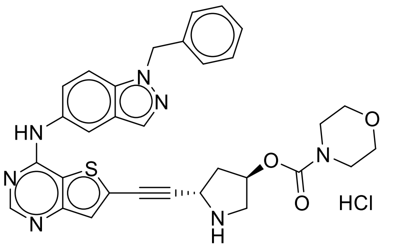	52	61	60
GSK182497A	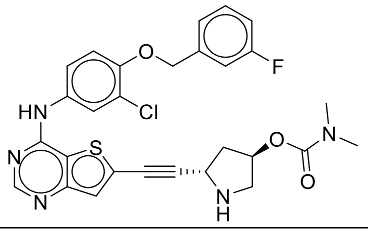	43	65	76

GSK192082A	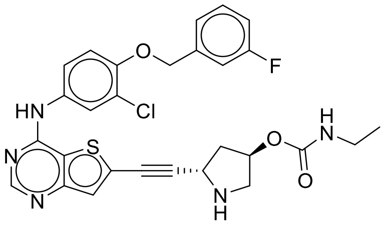	39	70	50
GW869810X	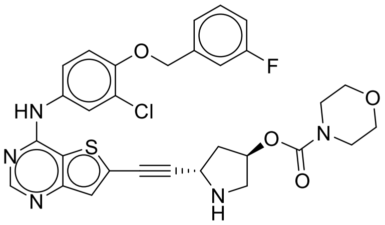	65	55	77
GW784684X	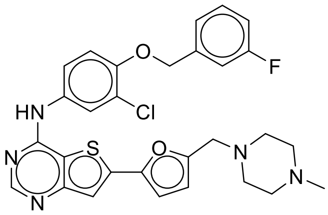	20	52	56
GSK300014A	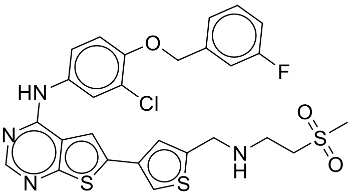	88	91	95
GW684626B	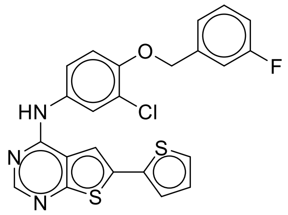	59	75	100
GW856804X	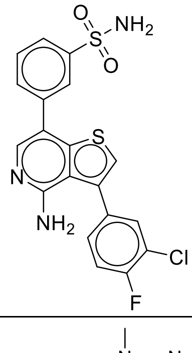	64	75	71
GW806742X	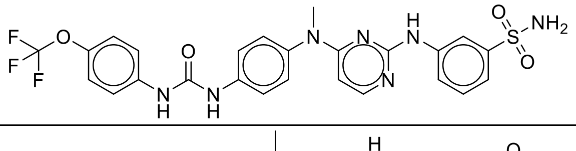	64	92	93
GW809897X	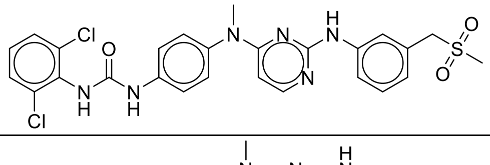	26	55	51
GW830900A	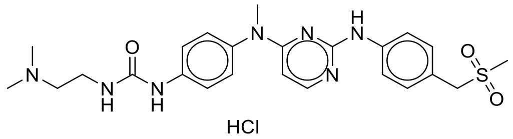	74	91	75

GW830263A	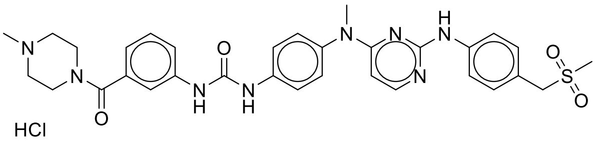	51	69	73
GW683134A	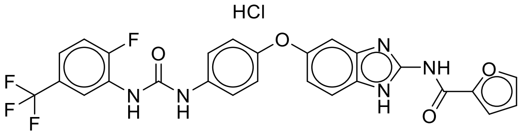	74	86	100
GW694590A	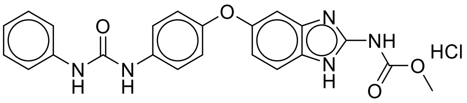	62	99	75
GW700494A	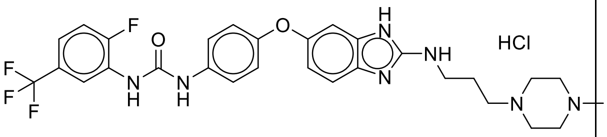	99	73	95
GW693917X	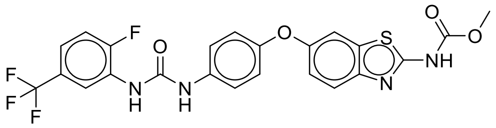	86	100	78
GW694234A	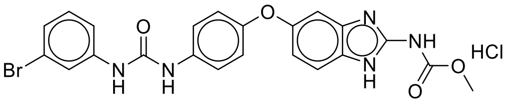	71	86	72
GW709042A	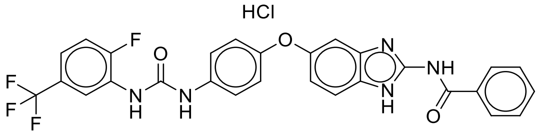	100	95	100
GW589961A	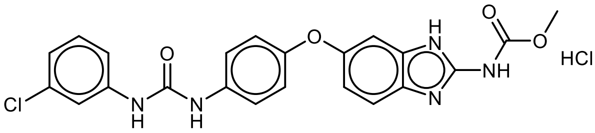	77	81	83
GW673715X	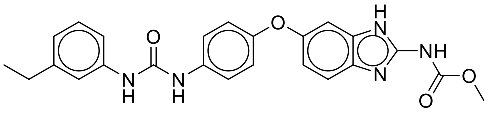	79	83	68
GW607049A	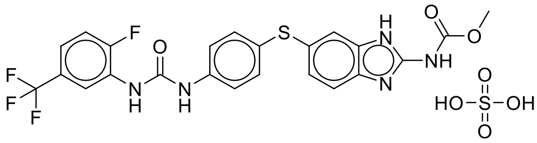	57	nd	70
GW607049C	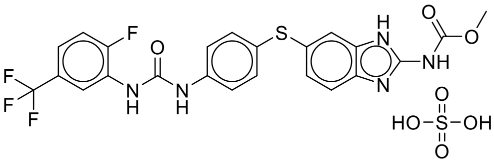	62	80	91
GSK718429A	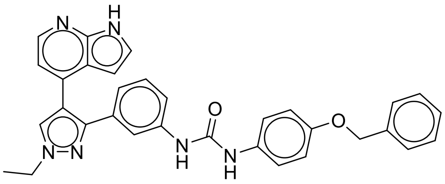	94	83	99

GW680908A	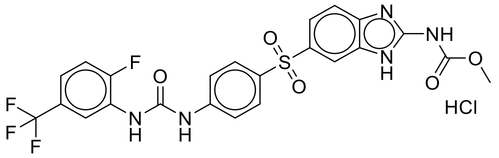	77	69	97
GSK237700A	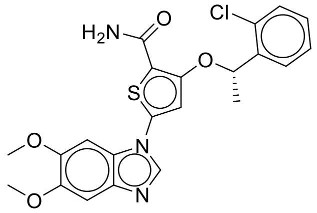	45	84	75
GSK319347A	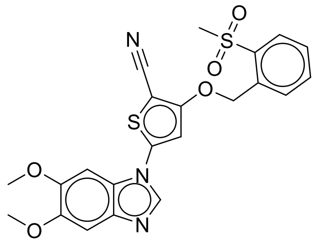	59	100	75
GSK1030058A	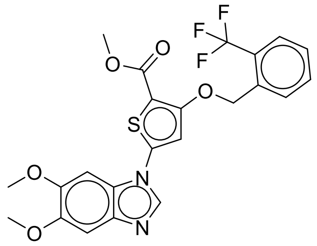	32	78	55
GW853606X	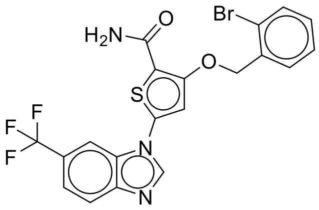	64	86	75
GSK312948A	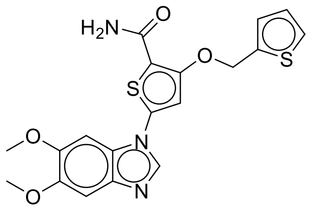	98	72	79
GSK571989A	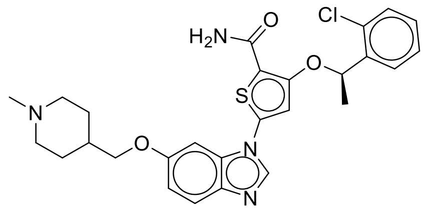	51	53	53
GW804482X	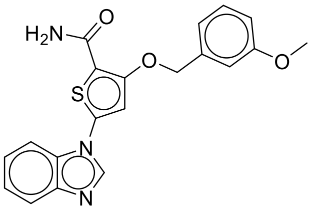	100	79	82

GSK1030062A	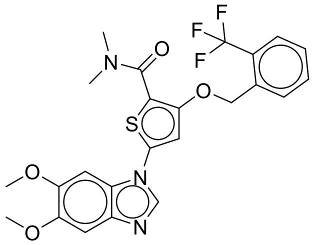	68	76	63
GSK1023156A	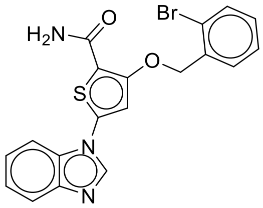	55	82	84
GW843682X	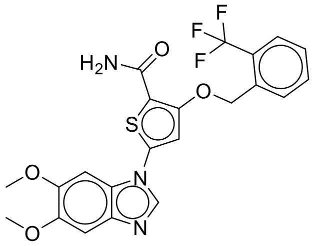	65	82	83
GSK204925A	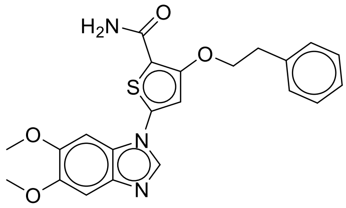	56	68	63
GSK237700A	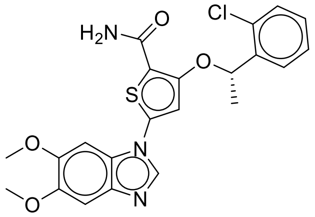	84	61	60
GSK317315A	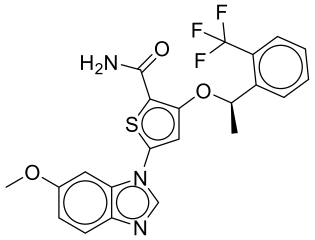	53	47	51
GW852849X	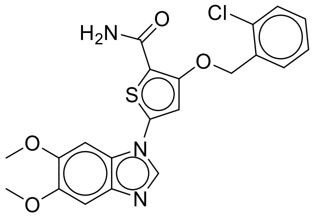	67	81	85
GSK579289A	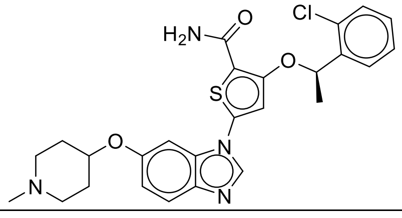	67	70	66

GW643971X	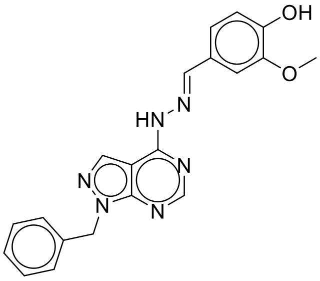	56	78	25
GW829874X	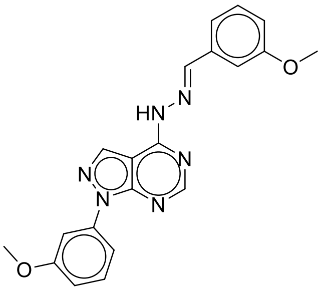	69	78	85
GW817396X	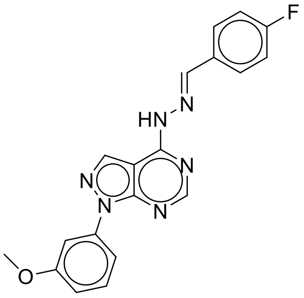	69	nd	41
GW643971X	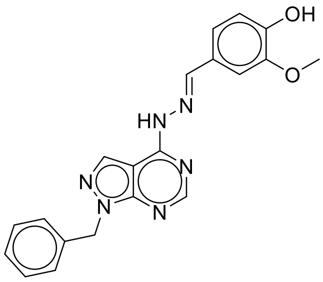	68	nd	27
GW829877X	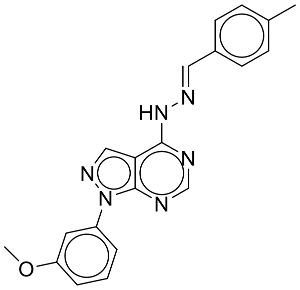	70	85	76
GW811168X	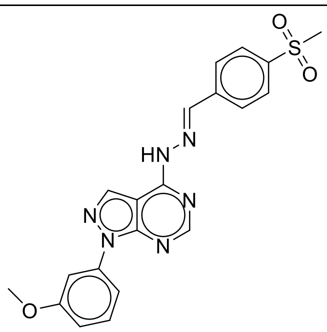	57	87	35

GW817394X	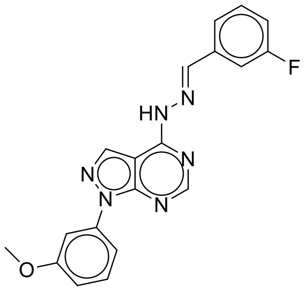	64	81	46
GW784752X	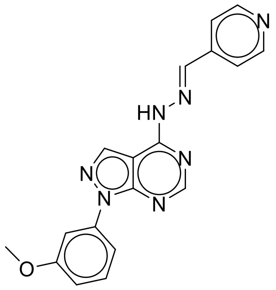	76	nd	93
GW811761X	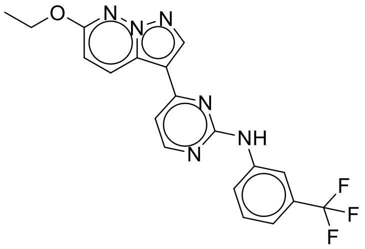	64	93	74
GW813360X	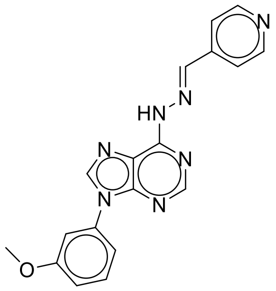	65	84	81
GW817396X	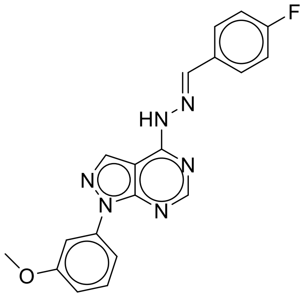	64	95	35
GW644007X	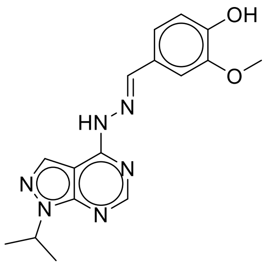	87	91	78

GW513184X	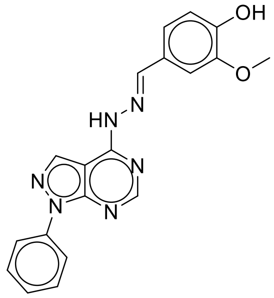	89	86	78
GW809885X	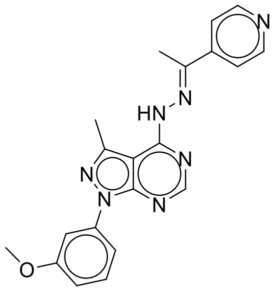	76	74	100
GW814408X	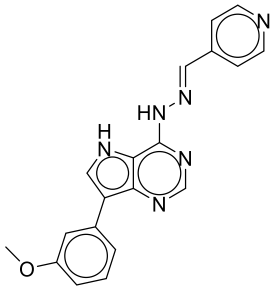	98	nd	87
SB-390527	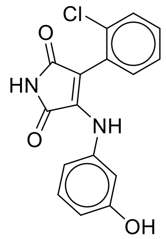	50	83	88
SB-390523	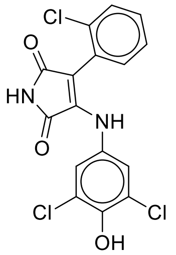	100	92	88
SB-358518	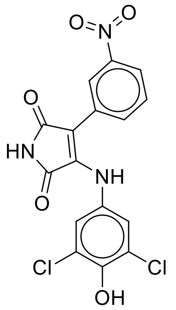	65	79	83

SKF-62604	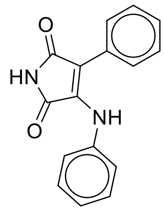	61	86	100
SB-361058	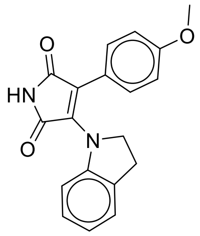	100	nd	97
SB-409513	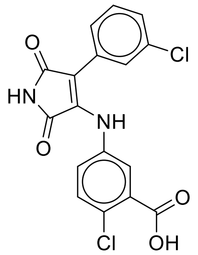	94	88	78
SB-333612	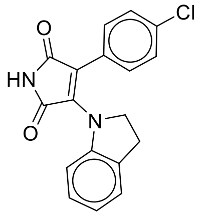	100	96	100
GW296115X	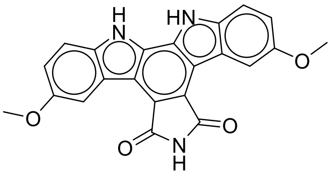	100	87	100
SB-376719	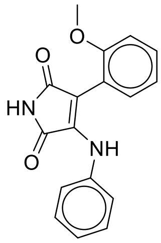	66	89	97
SB-333612	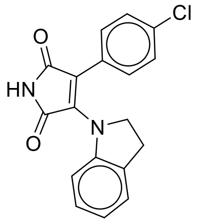	100	96	100
SB-360741	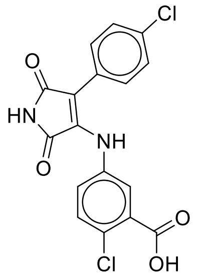	84	86	83

SB-361058	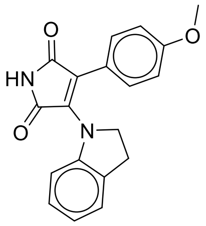	100	100	83
GW811761X	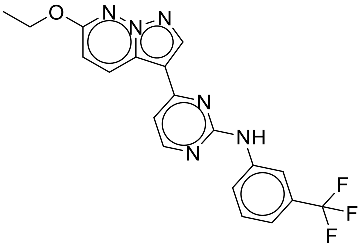	64	93	74
GW779439X	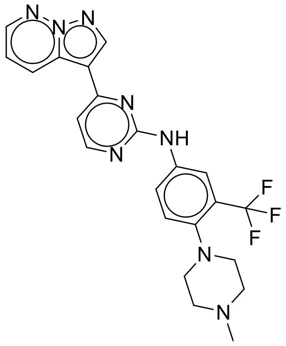	87	82	81
GW801372X	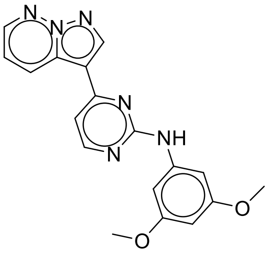	94	88	80
GW810372X	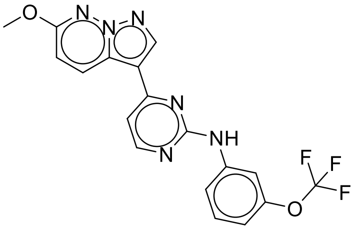	46	70	73
GW708336X	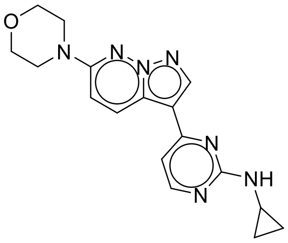	98	92	98
GW805758X	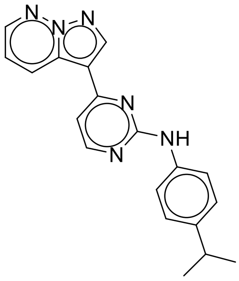	64	57	68

GW807982X	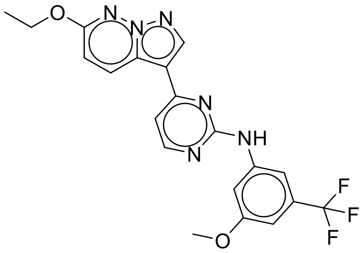	55	78	50
GW778894X	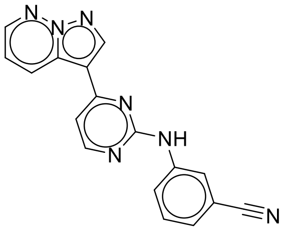	66	70	68
GW806290X	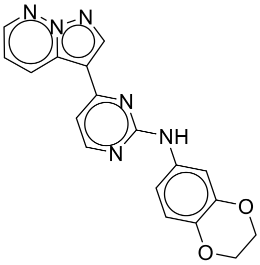	64	69	71
GW781673X	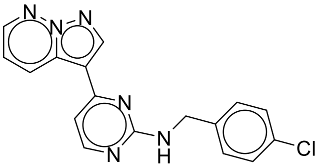	79	nd	63
GW780056X	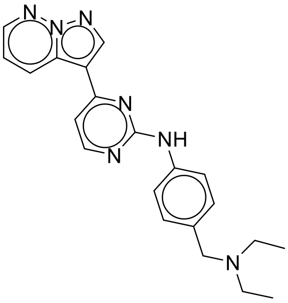	78	78	88
GW819077X	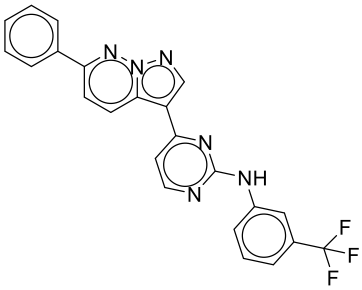	50	63	58
GW827396X	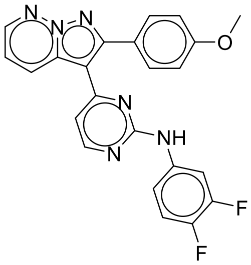	79%	61%	61%

GW827105X	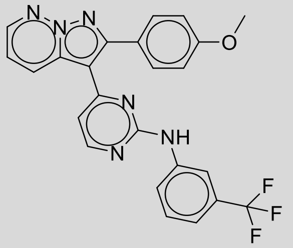	10% IC_{50} = 74 µM	80%	35%
GW827106X	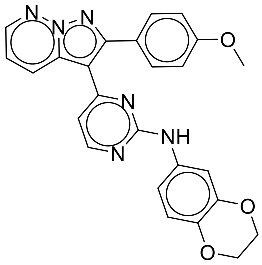	13	32	49
GW828525X	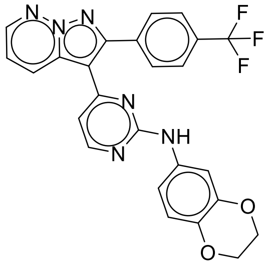	21	90	47
GW832467X	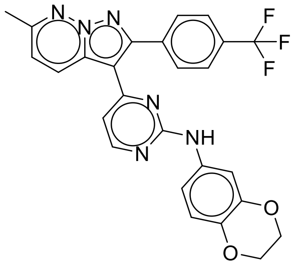	22	62	73
GSK2220400A	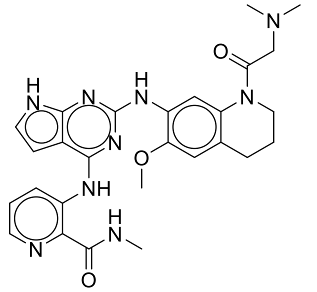	69	84	81
GSK2219385A	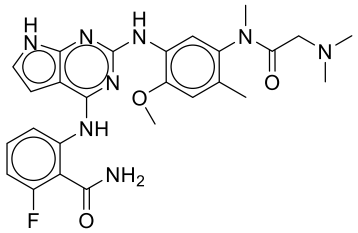	73	93	93
GSK1220512A	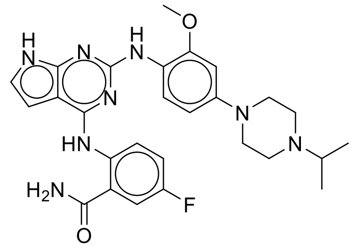	2	48	54

GSK1392956A	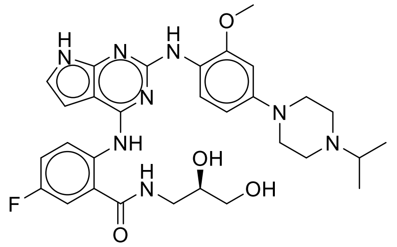	65	76	90
GSK1713088A	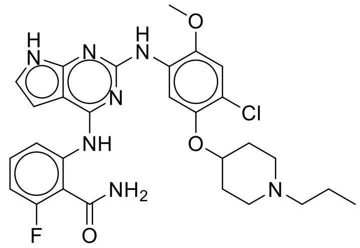	59	54	85
GSK1326255A	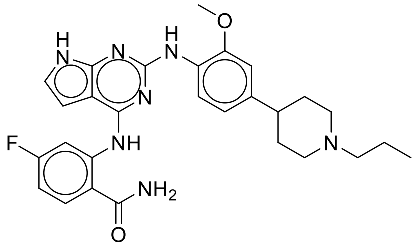	0	9<br>IC_{50}<br>= 39<br>μM	4
GSK1751853A	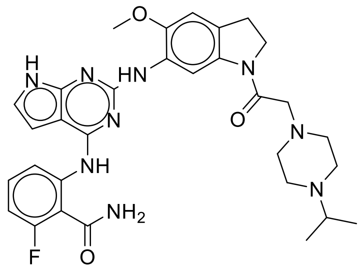	55	74	89
GSK2186269A	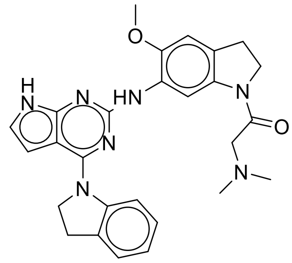	23	100	26
GSK1173862A	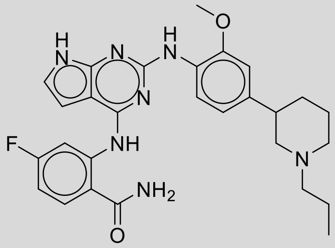	29	10<br>IC_{50}<br>= 32<br>μM	16
GSK1819799A	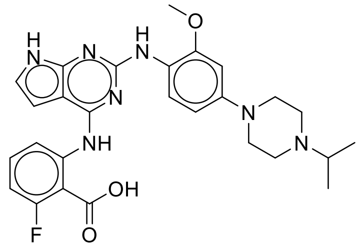	0	37	39

GW581744A	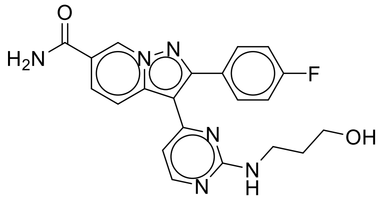	63	nd	83
GW569293E	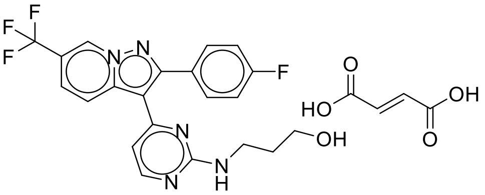	54	89	72
GW618013X	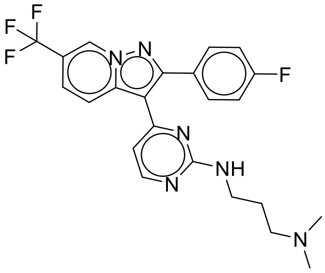	62	86	88
GW568326X	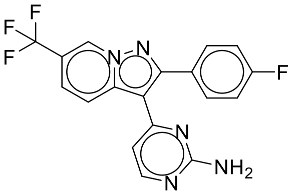	68	90	78
GW581744X	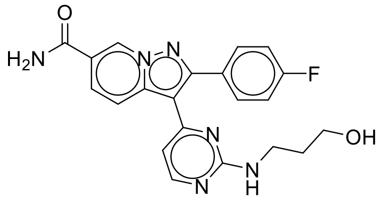	73	74	79
GW561436X	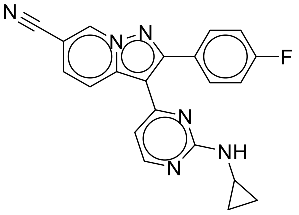	87	76	77
GW434756X	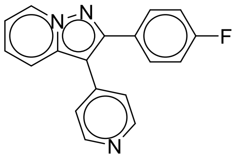	74	68	94
SB-400868-A	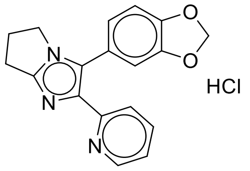	86	92	89
SB-698596-AC	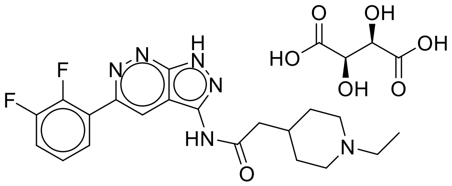	63	100	91

SB-675259-M	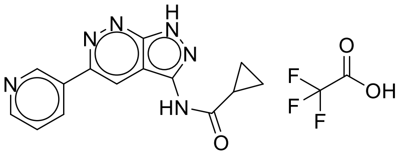	72	80	100
SB-686709-A	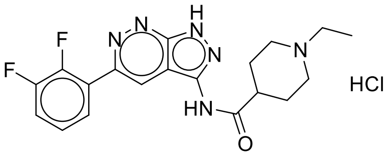	71	93	98
SB-678557-A	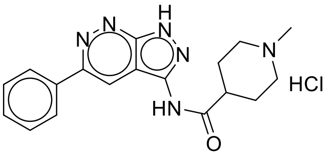	74	76	95
GW627512B	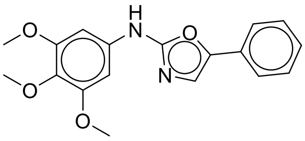	98	72	100
GW678313X	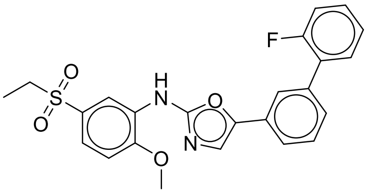	87	64	82
GW622055X	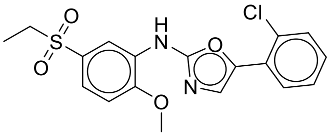	74	65	80
GW580509X	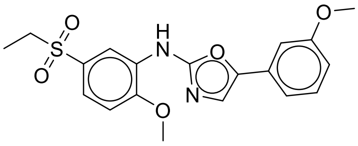	100	82	93
GW631581B	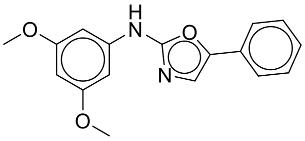	64	79	97
GW641155A	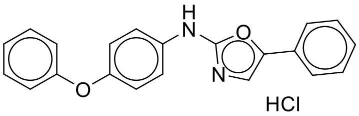	66	nd	86
GW549390X	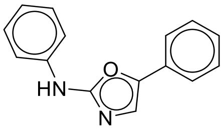	85	92	93
GW621970X	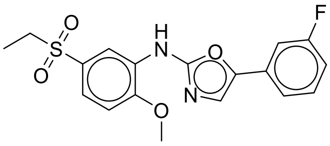	76	55	89
GW577921A	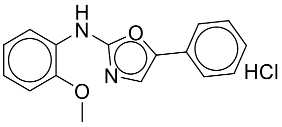	71	84	76

GW575533A	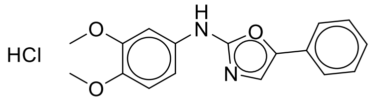	79	93	71
GW572399X	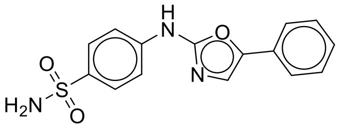	80	100	74
GW572401X	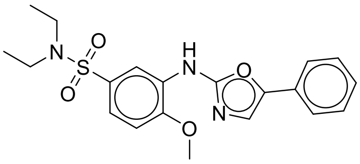	63	60	82
GW627834A	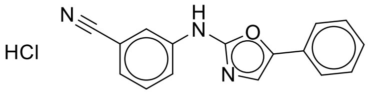	78	75	85
GW621431X	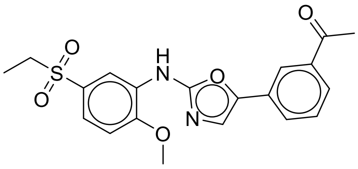	68	93	73
GW641155B	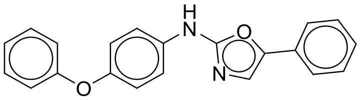	100	78	71
GW632046X	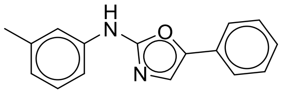	100	84	97
GW458344X	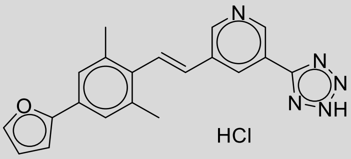	70%	45% IC_{50} = 104 μM	26 % IC_{50} = 59 μM
GW459057A	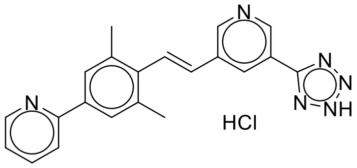	59	83	76
GW450241X	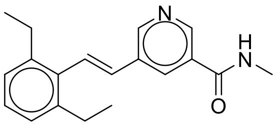	67	74	76
GW445017X	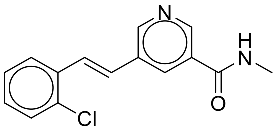	68	87	90
GW432441X	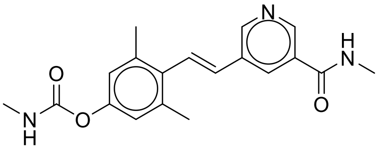	68	69	87
GW441806A	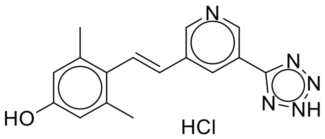	70	91	90

GW439255X	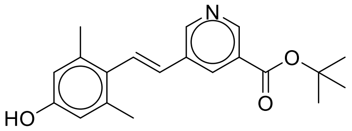	75	96	69
GW406731X	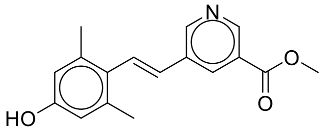	98	69	81
GW435821X	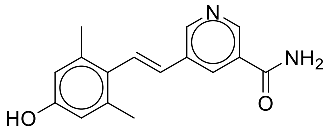	89	89	75
GW445012X	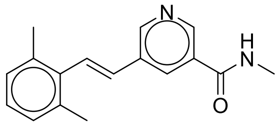	85	83	54
GW445014X	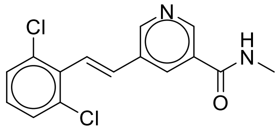	81	80	77
GW445015X	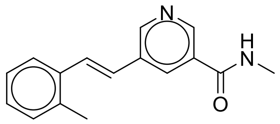	100	91	85
GW743024X	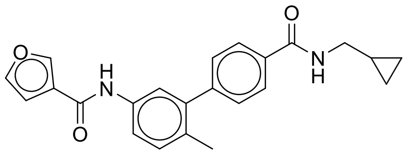	100	100	92
GW607117X	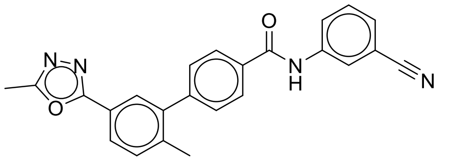	72	100	90
GW708893X	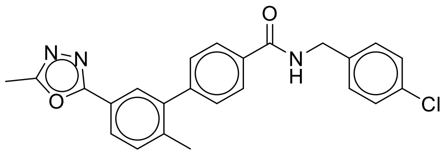	100	100	94
GW734508X	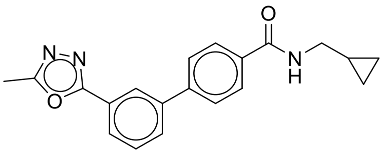	89	100	74
GW820759X	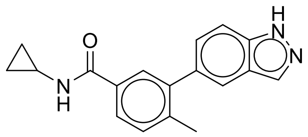	63	100	86
GW775608X	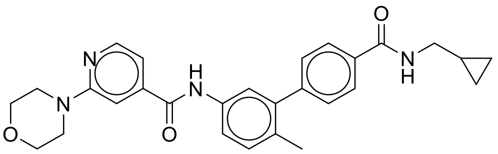	47	74	73

GW769076X	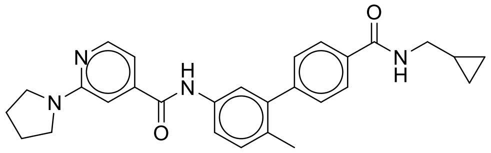	38	56	62
GSK635416A	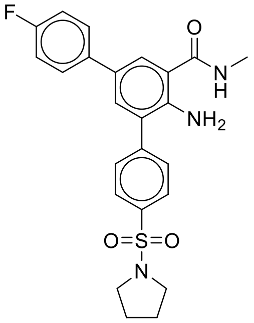	71	43	88
GW806776X	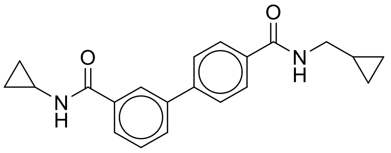	84	95	78
GSK711701A	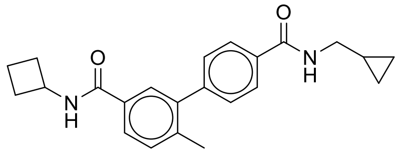	100	99	93
GW782912X	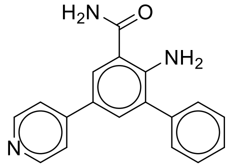	73	81	86
GSK625137A	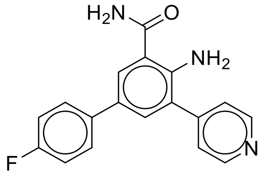	65	95	84
GSK620503A	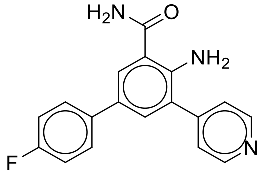	94	71	80
GSK605714A	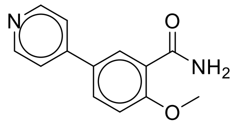	86	91	90
GW651576X	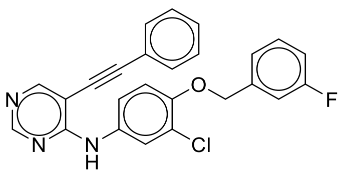	44% IC_{50} = 120 µM	43% IC_{50} = 90 µM	91
GW799251X	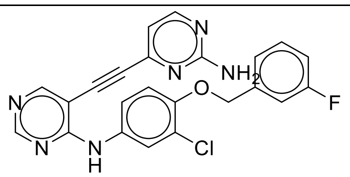	33	50	0

GW807930X	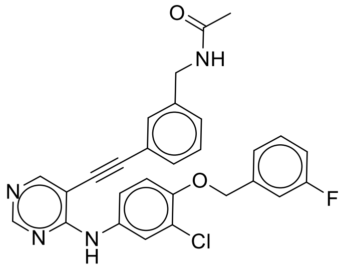	66	nd	69
GW659893X	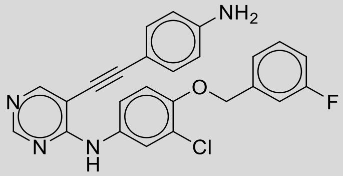	43% IC50 = 62 µM	51% IC50 = 104 µM	30% IC50 = 39 µM
SB-739245-AC	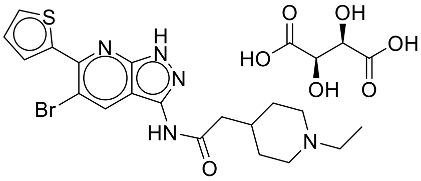	61	99	95
SB-739452	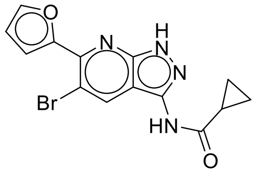	78	84	87
SB-743899	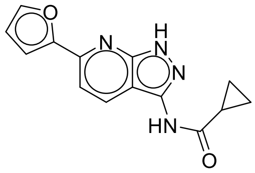	84	89	91
SB-732881	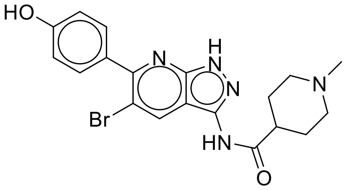	71	84	90
SB-725317	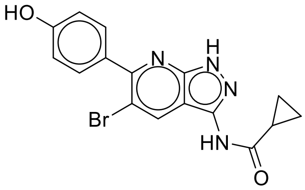	84	96	89
SB-735465	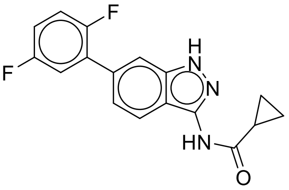	59	80	82
SB-741905	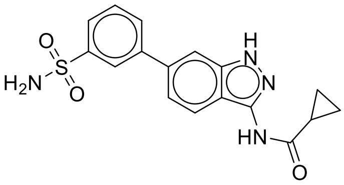	72	98	88

SB-742865	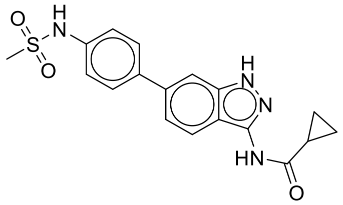	63	80	92
SB-735467	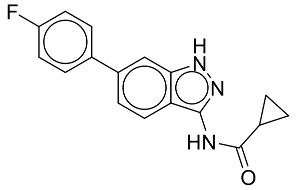	51	75	103
SB-738482	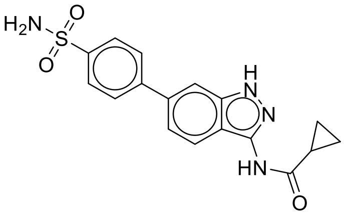	75	83	83
SB-742864	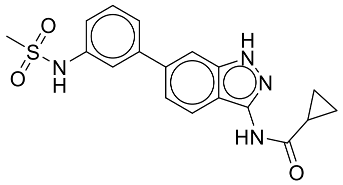	64	88	99
SB-732941	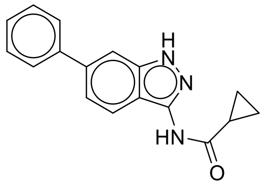	80	79	86
GW768505X	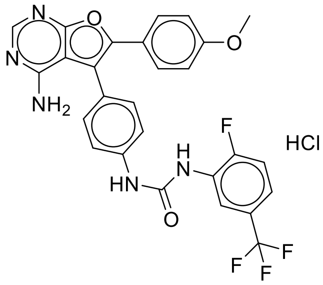	88	79	100
GW642125X	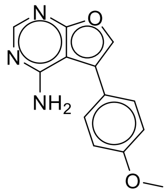	100	83	85
GW795486X	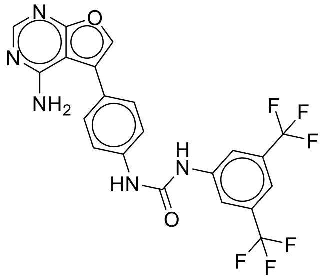	36	57	71

GW642138X	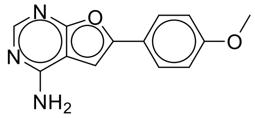	74	93	81
GW770249A	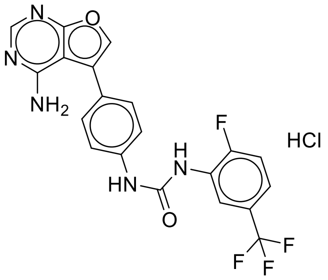	60	83	63
SB-814597	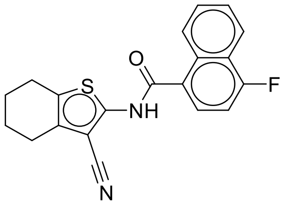	97	85	93
SB-347804	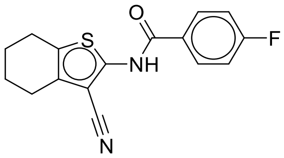	87	78	100
GW846105X	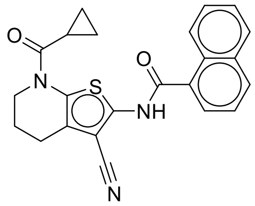	99	89	97
GW572738X	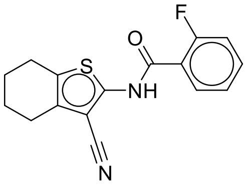	88	91	100
GW632580X	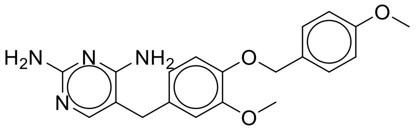	88	89	99
GW612286X	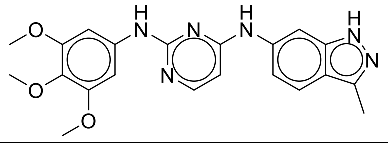	86	94	100
GW770220A	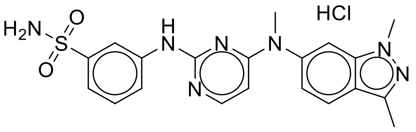	91	86	74
GW654652X	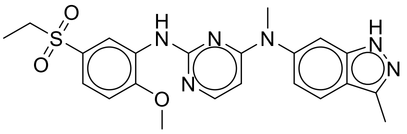	92	70	71

GW782612X	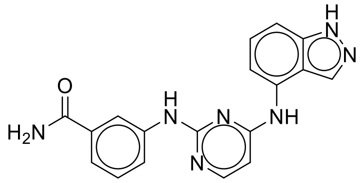	86	92	77
GW575808A	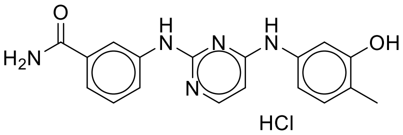	100	89	100
GW759710A	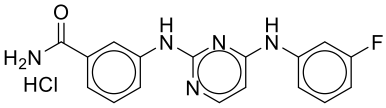	100	nd	100
GSK317354A	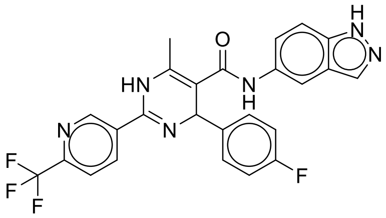	68	96	96
GSK466317A	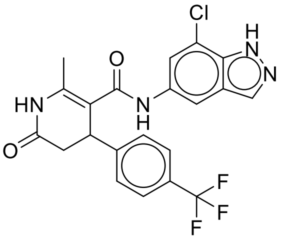	80	99	71
GSK299115A	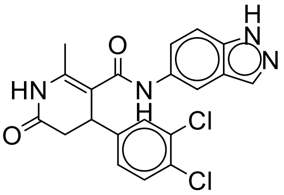	78	96	68
GSK180736A	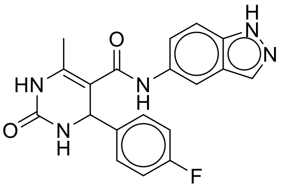	80	79	85
GSK466314A	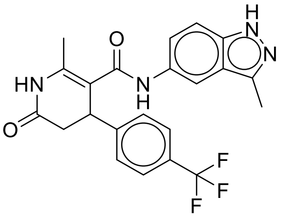	79	100	79
GSK270822A	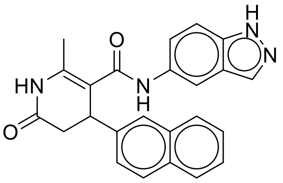	66	81	68

GW837331X	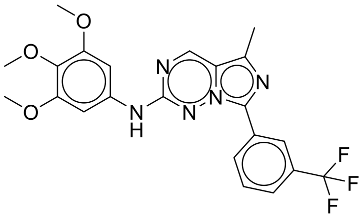	65	nd	89

nd… not determined due to lack of compound

2. Ligand efficiency values for hit compounds

Liganf afficinecy was calculated from established equations[1]:

$LE = (1.37 \times pIC_{50}) / HAC$

Where $pIC_{50} = \log_{10}(IC_{50} \times 10^{-x})$

(if IC_{50} = 1 µM, x = 6)

HAC = heavy atom count

Table S2: Ligand efficiency values for hit compounds

**Name**	**MW**	**IC_{50} MurC**	**IC_{50} MurD**	**IC_{50} MurE**	**IC_{50} MurF**	**LE (MurD)**	**LE (MurF)**
GW458344X	343.39	368 µM	104 µM	79 µM	59 µM	0.21	0.22
GW659893X	444.89	62 µM	104 µM	157 µM	39 µM	0.17	0.19
GW827105X	462.44	74 µM	83%	84%	100%	/	/
SB-242721	440.48	90 µM	63 µM	139 µM	95 µM	0.17	0.17
GSK1173862A	517.61	62 µM	32 µM	58 µM	66 µM	0.16	0.15

3. Enzyme kinetics graph:

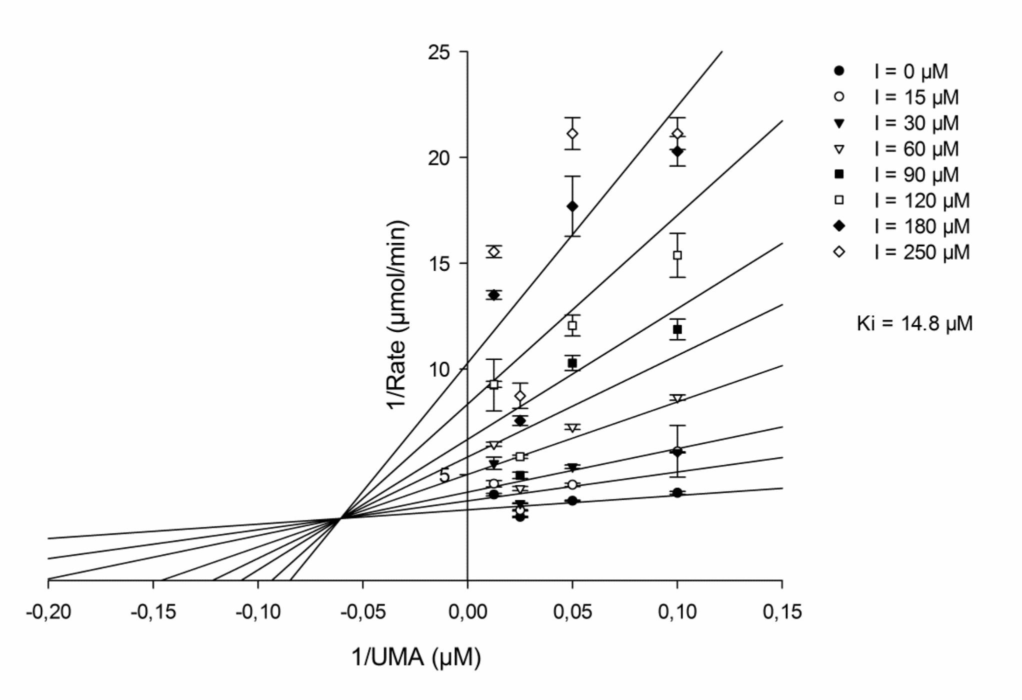

Figure S1. Lineweaver-Burk plot of mixed inhibition model of compound **1** *versus* uridine-5′-diphosphate-*N*-acetylmuramoyl-L-alanine at fixed ATP (400 µM) and D-Glu (100 µM).

4. Data of profiling in large panels of human kinase assays

To address the selectivity issue, all five hit compounds have been profiled in large panels of human kinase assays (over 200 kinase activity assays and an additional 68 differential scanning fluorimetry assays)). Only compound 5 (GSK1173862) shows appreciable activity at human kinases. The remaining four compounds have very little human kinase activity. Additional data for the NanoSyn panel are in the SI for PMID 26501955 (Jonathan M Elkins et al. Comprehensive characterization of the Published Kinase Inhibitor Set Nature Biotechnology volume 34, pages 95–103 (2016)).

Data in excel file: **PKIS kinase screening results**

5. Inhibitory activities of analogs of the compound1

Table S3: Results of in vitro biological assays of aza stilbene compounds against *E. coli* MurC, MurD and MurF ligases

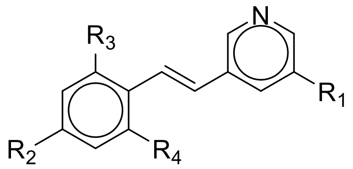

Compd	R_1	R_2	R_3	R_4	RA (%)[a] MurC	RA (%)[a] MurD	RA (%)[a] MurE	RA (%)[a] MurF
**1**	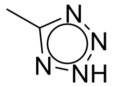	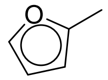	$-CH_3$	$-CH_3$	70	53	49	26
**6**	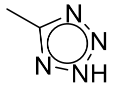	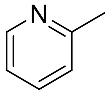	$-CH_3$	$-CH_3$	59	83	nd	76
**7**	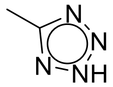	-OH	$-CH_3$	$-CH_3$	70	91	nd	90
**8**	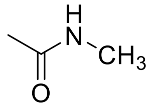	-H	-H	$-CH_3$	100	91	nd	85
**9**	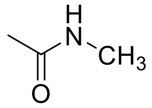	-H	$-CH_3$	$-CH_3$	85	83	nd	54
**10**	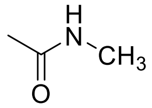	-H	-Cl	-Cl	81	80	nd	77
**11**	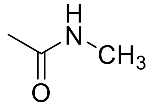	-H	-H	-Cl	68	87	nd	90
**12**	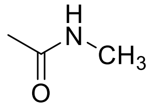	-H	-Et	-Et	67	74	nd	76
**13**	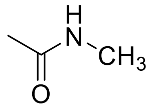	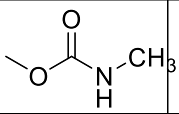	$-CH_3$	$-CH_3$	68	69	nd	87
**14**	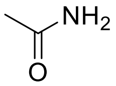	-OH	$-CH_3$	$-CH_3$	89	89	nd	75
**15**	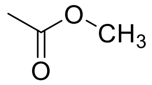	-OH	$-CH_3$	$-CH_3$	98	69	nd	81
**16**	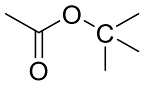	-OH	$-CH_3$	$-CH_3$	75	96	nd	69

nd… not determined

6. ChEMBL bioactivity search for hit compounds

Table S4: ChEMBL bioactivity search results for hit compounds

Comp.	Name	Structure	ChEMBLBioactivity Search Results
**1**	GW458344X	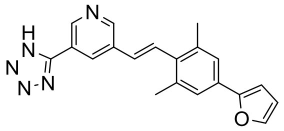	https://www.ebi.ac.uk/chembl/compound/inspect/CHEMBL373576
**2**	GW659893X	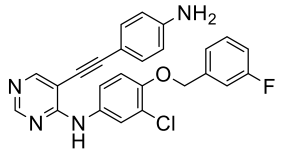	https://www.ebi.ac.uk/chembl/compound/inspect/CHEMBL205966
**3**	GW827105X	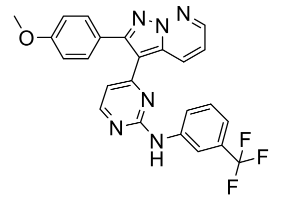	https://www.ebi.ac.uk/chembl/compound/inspect/CHEMBL186213
**4**	SB-242721	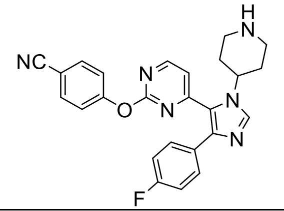	https://www.ebi.ac.uk/chembl/compound/inspect/CHEMBL13972
**5**	GSK1173862A	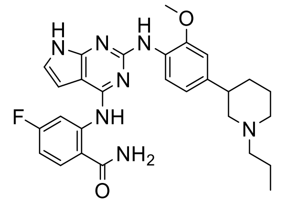	https://www.ebi.ac.uk/chembl/compound/inspect/CHEMBL477069

7. References:

1. Murray CW, Erlanson DA, Hopkins AL, Keserü GM, Leeson PD, Rees DC, Reynolds CH, Richmond NJ. Validity of ligand-efficiency metrics. ACS Med Chem Lett. 2014;5(6):616–618.
